# κ/β‐Ga_2_O_3_ Type‐II Phase Heterojunction

**DOI:** 10.1002/adma.202406902

**Published:** 2025-01-13

**Authors:** Yi Lu, Patsy A. Miranda Cortez, Xiao Tang, Zhiyuan Liu, Vishal Khandelwal, Shibin Krishna, Xiaohang Li

**Affiliations:** ^1^ Advanced Semiconductor Laboratory Electrical and Computer Engineering Program Division of Computer, Electrical, and Mathematical Sciences and Engineering (CEMSE) King Abdullah University of Science and Technology (KAUST) Thuwal 23955‐6900 Kingdom of Saudi Arabia

**Keywords:** DUV detection, gallium oxide (Ga_2_O_3_), phase heterojunction, self‐powered device, type‐II band alignment

## Abstract

Ultrawide‐bandgap gallium oxide (Ga_2_O_3_) holds immense potential for crucial applications such as solar‐blind photonics and high‐power electronics. Although several Ga_2_O_3_ polymorphs, i.e., α, β, γ, δ, ε, and κ phases, have been identified, the band alignments between these phases have been largely overlooked due to epitaxy challenges and inadvertent neglect. Despite having similar stoichiometry, heterojunctions involving different phases may exhibit band offsets. Here, β‐Ga_2_O_3_/κ‐Ga_2_O_3_‐stacked “phase heterojunction” is demonstrated experimentally. This phase heterojunction has a sharp and well‐defined interface, and subsequent measurements reveal an unbeknown type‐II band alignment with significant valence/conduction band offsets of ≈0.65 eV/0.71 eV. This alignment is promising for self‐powered deep ultraviolet (DUV) signal detection, necessitating an internal electric field near the junction and matching the absorption properties for effective electron–hole separation. The fabricated phase heterojunction photodetector displays a responsivity of three orders of magnitude higher at 17.8 mA W^−1^, with improved response times (rise time ≈0.21 s, decay time ≈0.53 s) under DUV illumination and without external bias in comparison to the bare β‐Ga_2_O_3_ and κ‐Ga_2_O_3_ photodetectors, confirming the strong interfacial electrical field. This study provides profound insight into Ga_2_O_3_/Ga_2_O_3_ heterojunction interfaces with different polymorphs, allowing the use of phase heterojunctions to advance electronic device applications.

## Graphical abstract

Researchers have experimentally unveiled a perfect interface between the monoclinic (β)‐phase and orthorhombic (κ)‐phase of Ga_2_O_3_, establishing a “phase heterojunction” within the ultrawide bandgap Ga_2_O_3_ material system. This phase heterojunction exhibits a type‐II band alignment with significant band offset, highlighting a large interfacial electrical field, which is verified through the separation of photogenerated electron–hole pairs, even without external bias.

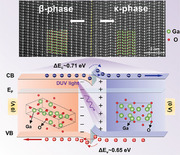

## 1 Introduction

Ultrawide‐bandgap (UWBG) gallium oxide (Ga_2_O_3_) is emerging as a promising candidate for advanced electronic applications, including solar‐blind photonics and high‐power electronics. This is attributed to its wide bandgap (≈5 eV), reasonably high electron mobility, and high critical breakdown field (≈8 MV cm^−1^).^[^ ^1^ ^]^ Compared with other semiconductors, such as SiC and GaN, Ga_2_O_3_ has a wider bandgap, lower substrate cost, higher breakdown field strength, and higher Baliga figure of merit.^[^ ^2^ ^]^ Additionally, when compared to other UWBG materials such as AlN and diamond, Ga_2_O_3_ offers advantages in mature material synthesis and device fabrication.^[^ ^3^ ^]^

Ga_2_O_3_ exhibits several identified polymorphs, including corundum (α), monoclinic (β), cubic defect spinel (γ), bixbyite (δ), hexagonal (ε), and orthorhombic (κ) phases.^[^ ^4^ ^]^ Among these, β‐phase Ga_2_O_3_ with monoclinic structure has received the most significant attention due to its thermodynamic stability and availability of native substrates. Various applications of deep ultraviolet (DUV) detectors, Schottky diodes, and field‐effect transistors have been realized using epitaxial β‐phase Ga_2_O_3_ thin films.^[^ ^5^ ^]^ Furthermore, γ‐Ga_2_O_3_ and δ‐Ga_2_O_3_ show promise in applications, such as spintronics and power electronics,^[^ ^6^ ^]^ while they have been much less thoroughly investigated, partially due to the associated experimental challenges in the material synthesis. The ε‐phase Ga_2_O_3_ is noted for its large polarization constant among the different polymorphs, making it a promising choice for polarization engineering applications.^[^ ^7^ ^]^ Furthermore, α‐Ga_2_O_3_ can be synthesized on *m*‐plane sapphire substrates using physical vapor deposition and chemical vapor deposition methods. Its substantial bandgap energy (≈5.3 eV) offers considerable flexibility in band engineering.^[^ ^8^ ^]^ Furthermore, in recent years, the use of Sn as a catalyst to induce κ‐phase Ga_2_O_3_ during physical deposition, termed as metal‐oxide‐catalyzed epitaxy, has been successfully demonstrated in several studies. This approach has enabled the production of high quality κ‐Ga_2_O_3_ thin film membranes and high‐performance DUV photodetectors (PDs).^[^ ^9^ ^]^ The crystal structures and parameters of abovementioned Ga_2_O_3_ polymorphs are summarized in Table S1 (Supporting Information).

Similar to Ga_2_O_3_, various other materials inherently exhibit multiple crystallographic phases, each with distinct structural characteristics. Differences in phase within nominally the same material result in significant variations in the physical and chemical properties, affecting the carrier mobility, chemical stability, energy bandgap, and more. Furthermore, a number of studies have reported successful formation of “phase heterojunction (PHJ)”, which involves junctions between different phases of the same material. Examples of such PHJ include wurtzite/zincblende III‐nitride,^[^ ^10^ ^]^ rutile TiO_2_/anatase TiO_2_,^[^ ^11^ ^]^ α‐Bi_2_O_3_/β‐Bi_2_O_3_,^[^ ^12^ ^]^ 0D Bi_4_MoO_9_ quantum dots /2D Bi_2_MoO_6_ nanosheets,^[^ ^13^ ^]^ α‐CdS/β‐CdS,^[^ ^14^ ^]^ wurtzite InP/zincblende InP,^[^ ^15^ ^]^ and γ‐CsPbI_3_/β‐CsPbI_3_.^[^ ^16^ ^]^ These studies consistently demonstrated a staggered type‐II band alignment at the PHJ, where the internal electric field significantly enhanced the electron–hole separation efficiency. For example, investigations of heterojunctions, such as zincblende–GaN/InN and zincblende–AlN/GaN, revealed distinct differences in the bandgap and electron affinity when compared to their wurtzite‐phase counterparts, namely, wurtzite–GaN/InN and wurtzite–AlN/GaN heterojunctions, respectively.^[^ ^10b^ ^]^ Additionally, a type‐II staggered band alignment of ≈0.4 eV offset has been confirmed between anatase TiO_2_ and rutile TiO_2_.^[^ ^11a,b^ ^]^ This alignment facilitates the robust separation of photoexcited charge carriers between the two phases, providing an effective strategy for enhancing photocatalytic efficiency. Moreover, solar cells incorporating γ‐CsPbI_3_/β‐CsPbI_3_ perovskite PHJ benefit from an increased built‐in potential between the two phases, achieving a high power conversion efficiency of 21.5%.^[^ ^16^ ^]^ These PHJs demonstrate their significance in enhancing the device performance across various applications, including solar cells, photocatalysis, transistors, water splitting, and PDs, as detailed in Table S2 (Supporting Information).

Therefore, extending the scope beyond the individual phases of Ga_2_O_3_, the integration of the aforementioned diverse Ga_2_O_3_ phases (α, β, γ, δ, ε, and κ) to form what is termed as Ga_2_O_3_/Ga_2_O_3_ PHJ may potentially yield unique junction properties arising from variations in bandgap or electron affinity between each Ga_2_O_3_ phase. For example, theoretic calculation shows a considerable band offset between β and α phases of Ga_2_O_3_, and another theoretic study reported a type‐II band alignment with a valance band of α‐Ga_2_O_3_, which is 0.35 eV higher than that of β‐Ga_2_O_3_.^[^ ^17^ ^]^ However, direct experimental evidence is lacking. Furthermore, some studies have reported Ga_2_O_3_/Ga_2_O_3_ junctions via the annealing of one metastable phase (e.g., α or γ phase) to partially convert it into the most‐thermally stable β phase,^[^ ^18^ ^]^ which, however, form randomly distributed mixed phases with multiple crystal orientations and unclear interfaces. Moreover, in our previous studies, a type‐II alignment was observed between the β‐Ga_2_O_3_ and AlN.^[^ ^19^ ^]^ Our further experimental results confirmed a type‐I alignment between κ‐Ga_2_O_3_ and AlN when using the pulsed laser deposition (PLD) growth method on the same AlN template.^[^ ^20^ ^]^ Consequently, a potential band offset is anticipated at the β‐Ga_2_O_3_/κ‐Ga_2_O_3_ junction, implying a type‐II band alignment. For a long time, the band offset and alignment of this PHJ have been typically overlooked because they have similar stoichiometry and epitaxy challenges. The formation of this PHJ may hold significant potential for a variety of electronic and optoelectronic applications, where a type‐II junction is desired for the efficient separation of photogenerated carriers,^[^ ^21^ ^]^ as depicted in various PHJ devices in Table S2 (Supporting Information). However, it is worth noting that concrete experimental evidence to determine the band alignment of the Ga_2_O_3_ PHJ remains vague, and the electrical properties of this junction have not been previously examined due to the epitaxy challenge of achieving a distinct PHJ interface.

Therefore, in this study, we demonstrate β‐phase/κ‐phase‐stacked Ga_2_O_3_ PHJ, which features a type‐II band alignment, creating a depletion region for efficient electron–hole separation and enabling self‐powered DUV detection. The motivations for studying β/κ‐Ga_2_O_3_ PHJ include the following: the capability to achieve high‐quality epitaxy, the superior performance of discrete devices fabricated from these materials, and the potentially staggered band alignment, as suggested by our previous reports.^[^ ^9^, ^20^, ^22^ ^]^ The clear interface between the β‐phase and κ‐phase Ga_2_O_3_ is confirmed via element distributions and atomic level arrangement of atoms, revealing a high quality semiconductor heterojunction, which comprises a sharp interface between two distinctive Ga_2_O_3_ phases. Moreover, an unbeknown type‐II band alignment with significant valence/conduction band offsets of ≈0.65/0.71 eV between the β‐phase and κ‐phase Ga_2_O_3_ is revealed through photoelectron spectroscopy. The establishment of a type‐II band alignment results in an interfacial electrical field, which is validated by comparing the junction's DUV photoresponse to that of bare β‐phase Ga_2_O_3_ and κ‐phase Ga_2_O_3_. All the presented results demonstrate the type‐II alignment of β/κ‐Ga_2_O_3_ PHJ and its application potentials.

## 2 Result and Discussion

**Figure 1:**
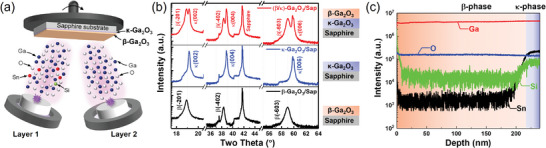
a) Schematic figure of β‐phase/κ‐phase Ga_2_O_3_ PHJ growth. b) X‐ray diffraction (XRD) patterns of β‐phase Ga_2_O_3_, κ‐phase Ga_2_O_3_, and β‐phase/κ‐phase Ga_2_O_3_ grown on sapphire substrate. c) Secondary ion mass spectrometry (SIMS) profile distribution of Ga, O, Si, and Sn elements of β‐phase/κ‐phase Ga_2_O_3_ sample.

**Figure** **1**a shows the β‐Ga_2_O_3_/κ‐Ga_2_O_3_ PHJ growth process. The detailed growth conditions can be found in the Experimental Section. Figure 1b presents the X‐ray diffraction (XRD) patterns of the grown β‐phase/κ‐phase Ga_2_O_3_ sample, along with two control samples: bare β‐phase Ga_2_O_3_ and bare κ‐phase Ga_2_O_3_ grown on sapphire substrates. A noticeable difference in the XRD patterns of orthorhombic κ‐Ga_2_O_3_ and monoclinic β‐Ga_2_O_3_ is evident. Specifically, κ‐Ga_2_O_3_(002), (004), and (006) exhibit shifts of ≈+0.26°, +0.52°, and +0.79° relative to β‐Ga_2_O_3_(−201), (−402), and (−603), respectively. Furthermore, XRD asymmetric phi scan performed for (122) and (131) reflection of bare κ‐Ga_2_O_3_ film confirms its orthorhombic structure, as shown in Figure S1a (Supporting Information). The κ‐Ga_2_O_3_/sapphire sample displays narrower peaks when compared to β‐Ga_2_O_3_/sapphire, indicating better material crystallinity, which can also be viewed from the XRD rocking curve (Figure S1b, Supporting Information). This is due to the fact that κ‐Ga_2_O_3_/sapphire exhibits smaller lattice mismatch (≈4.1%) than β‐Ga_2_O_3_/sapphire (≈6.6%).^[^ ^23^ ^]^ In the case of the β‐phase/κ‐phase Ga_2_O_3_ sample, the XRD pattern reveals the presence of κ‐Ga_2_O_3_(002), (004), (006) and β‐Ga_2_O_3_(−201), (−402), (−603) peaks. Importantly, a distinct separation between the positions of the κ‐phase and β‐phase peaks is observed, which is consistent with the stacked structure of the β‐phase/κ‐phase Ga_2_O_3_. The lattice mismatch between β‐Ga_2_O_3_ and κ‐Ga_2_O_3_ is calculated to be ≈4.82% based on the crystal model along the growth direction, as shown in Figure S2 (Supporting Information).

In Figure 1c, the element distributions obtained by secondary ion mass spectrometry (SIMS) for the grown β‐phase/κ‐phase Ga_2_O_3_ sample are displayed. Notably, the intensities of Sn and Si exhibit a pronounced increase at the interface between β‐phase and κ‐phase Ga_2_O_3_. This phenomenon can be attributed to the presence of Si and Sn in the target material, specifically Ga_2_O_3_:SnO_2_:SiO_2_ (98.4:1.5:0.1, wt%), used during the deposition of κ‐phase Ga_2_O_3_. The incorporation of Sn serves a dual purpose. It aids in the formation of Ga_2_O_3_ by reducing suboxide (Ga_2_O) etching under high vacuum ambient, and it occupies octahedral lattice sites for facilitating κ‐phase synthesis.^[^ ^24^ ^]^ Detailed synthesis mechanisms are shown in Figures S3 and S4 (Supporting Information). Moreover, Si is incorporated because of its amphoteric behavior in Ga_2_O_3_, particularly in κ‐Ga_2_O_3_.^[^ ^25^ ^]^ For comparison, SIMS and *IV* curves of κ‐phase Ga_2_O_3_ synthesized with and without Si incorporation are provided in Figure S5 (Supporting Information), illustrating that Si acts as an acceptor (or compensator) rather than a donor in κ‐Ga_2_O_3_, resulting in a highly insulating property of κ‐Ga_2_O_3_. Additionally, in a previous study, intentional doping with Si led to a compensation effect, effectively reducing the dark current.^[^ ^9d^ ^]^ The distinct differences in Si and Sn intensities confirm the presence of a well‐defined interface between β‐phase and κ‐phase Ga_2_O_3_.

**Figure 2:**
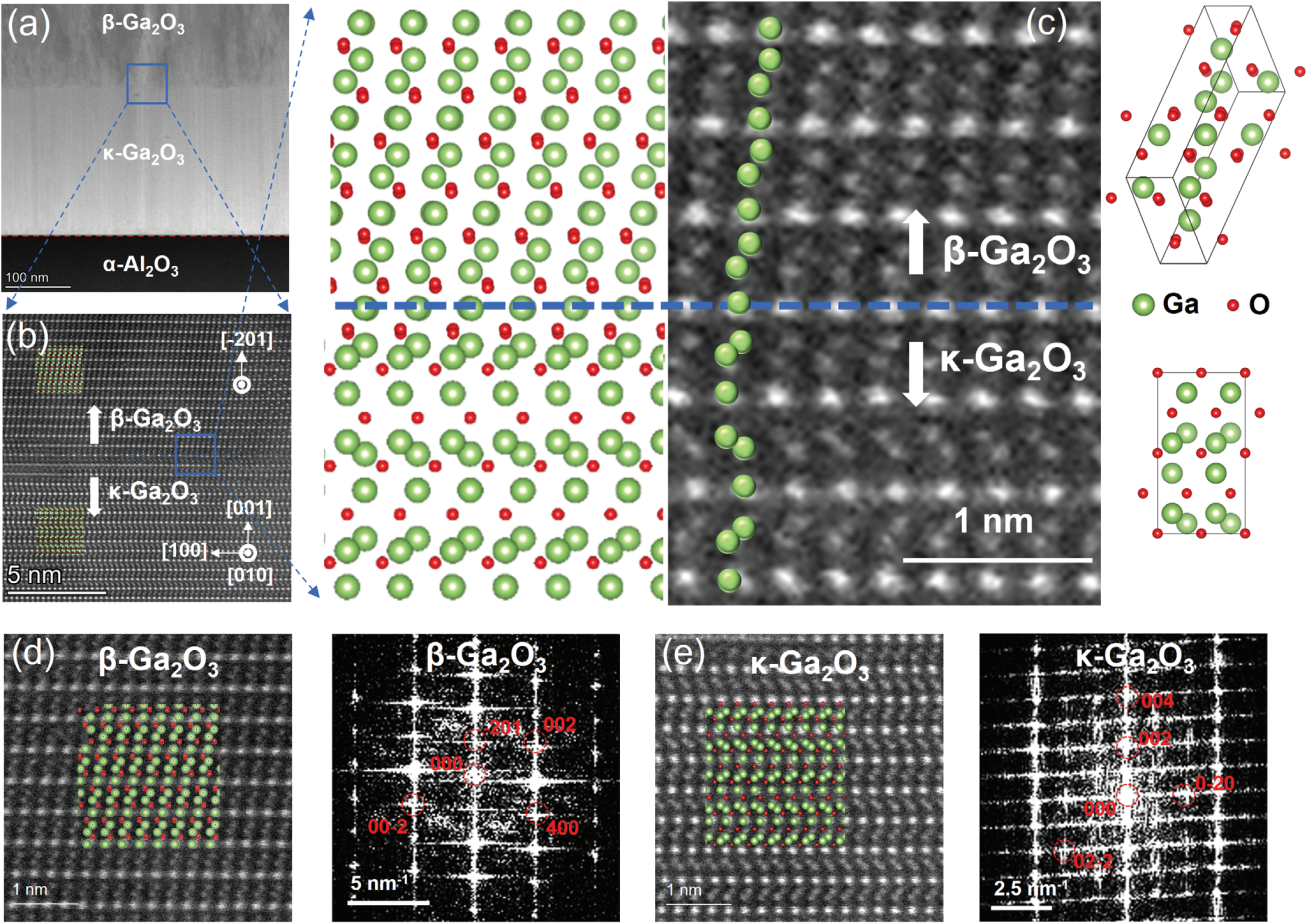
a) Large‐scale cross‐sectional transmission electron microscopy (TEM) of β‐phase/κ‐phase Ga_2_O_3_ grown on sapphire substrate. b) High‐resolution TEM (HR‐TEM) image of β‐phase/κ‐phase Ga_2_O_3_ interface. c) Enlarged view of the β‐Ga_2_O_3_ and κ‐Ga_2_O_3_ interface with atomic models overlaid, illustrating the clear, sharp, and well‐defined interface. Atomic arrangement and fast Fourier transform (FFT) images of d) β‐Ga_2_O_3_ and e) κ‐Ga_2_O_3_.

In **Figure** **2**a, a large‐scale cross‐section transmission electron microscopy (TEM) image of the β‐phase/κ‐phase Ga_2_O_3_ film is presented. Furthermore, ≈200 nm κ‐Ga_2_O_3_ thin film exhibits a distinct interface with the sapphire substrate, and on top of that, β‐Ga_2_O_3_ is epitaxially grown. Figure 2b displays a high‐resolution TEM (HR‐TEM) image of κ‐Ga_2_O_3_ and β‐Ga_2_O_3_, clearly revealing the atomic arrangement and their sharp interface (as shown using the blue dashed curve). Figure 2c displays an enlarged view of the β‐Ga_2_O_3_ and κ‐Ga_2_O_3_ interface with atomic models overlaid. A crystal model of the monoclinic β‐Ga_2_O_3_ structure (space group #12, *C*2*/m*)^[^ ^26^ ^]^ along [−201] growth direction aligns well with the crystal lattice observed in the HR‐TEM image. As for κ‐Ga_2_O_3_, the crystal model for the orthorhombic structure (space group #33, *Pna*2_1_)^[^ ^27^ ^]^ along [010] zone axis also corresponds with the HR‐TEM pattern. The interface is distinct and accurately mapped with atomic models, representing an identified β/κ‐Ga_2_O_3_ PHJ with the smooth transition from κ‐phase to β‐phase. Figure 2d,e presents the HR‐TEM image and the corresponding fast Fourier transform (FFT) images for β‐Ga_2_O_3_ and κ‐Ga_2_O_3_, respectively. These images reveal labeled diffraction spots, such as κ‐Ga_2_O_3_(002), (004), (0−20), and (02−2), as well as β‐Ga_2_O_3_(−201), (002), and (400) diffraction spots. The epitaxial relationship is <001> κ‐Ga_2_O_3_ || <−201> β‐Ga_2_O_3_, consistent with the XRD 2*θ*–*ω* profile shown in Figure 1b. The clear experimental atomic arrangement and its epitaxial relationship shown in this study can be leveraged by the research community to further explore atomic‐scale interactions along β/κ‐Ga_2_O_3_ interface using theoretic calculations.

In general, X‐ray photoelectron spectroscopy (XPS) is a commonly used technique for determining the band offset in various heterojunctions. XPS enables direct measurements of core‐level and valence‐edge energies, which can be utilized to calculate the Δ*E*_v_. This calculation is often performed using Equation (1) and is commonly referred to as the Kraut method:^[^ ^34^ ^]^

(1) $$\Delta E_{v}=\left(E_{\mathrm{CL}}^{Y}-E_{\mathrm{VBM}}^{Y}\right)-\left(E_{\mathrm{CL}}^{X}-E_{\mathrm{VBM}}^{X}\right)-{\left(E_{\mathrm{CL}}^{Y}-E_{\mathrm{CL}}^{X}\right)}_{\mathrm{interface}}$$

where *E*_CL_ is chosen as the core levels of each material *X* and *Y*. This equation combines measurements from “bulk” samples of *X* and *Y* (the first four terms) and measurements from an “interface” sample (the last two terms) to determine the Δ*E*_v_ between these materials. The method also considers band bending due to interface states and other factors.

However, the Kraut method is only applicable when the measurement can distinguish between the core levels of the two materials. In cases where the interface involves a semiconductor and its oxide, or a junction containing the same atoms, Equation (1) can be simplified to a more straightforward expression

(2) $$\Delta E_{v}\approx \left(E_{\mathrm{VBM}}^{X}-E_{\mathrm{VBM}}^{Y}\right)$$

In this case, the valence band offset can be approximated by considering the difference between the valence band maxima (VBM) of the two materials. This simplification was based on the principle that when *X* and *Y* are in physical contact, their Fermi levels are aligned. This method has been effectively applied in the study of interfaces such as the Si/SiO_2_ interface^[^ ^35^ ^]^ and BGaN/GaN.^[^ ^36^ ^]^

Additionally, Δ*E*_c_ of the junction can be determined using the Δ*E*_v_ results and the bandgap of the materials as follows

(3) $$\Delta E_{c}=E_{g}^{Y}-E_{g}^{X}+\Delta E_{v}$$

where $E_{g}^{Y}\;$ and $E_{g}^{X}\;$ represent the bandgaps of materials *Y* and *X*, respectively. These bandgap values were obtained through UV–vis measurements.

**Figure 3:**
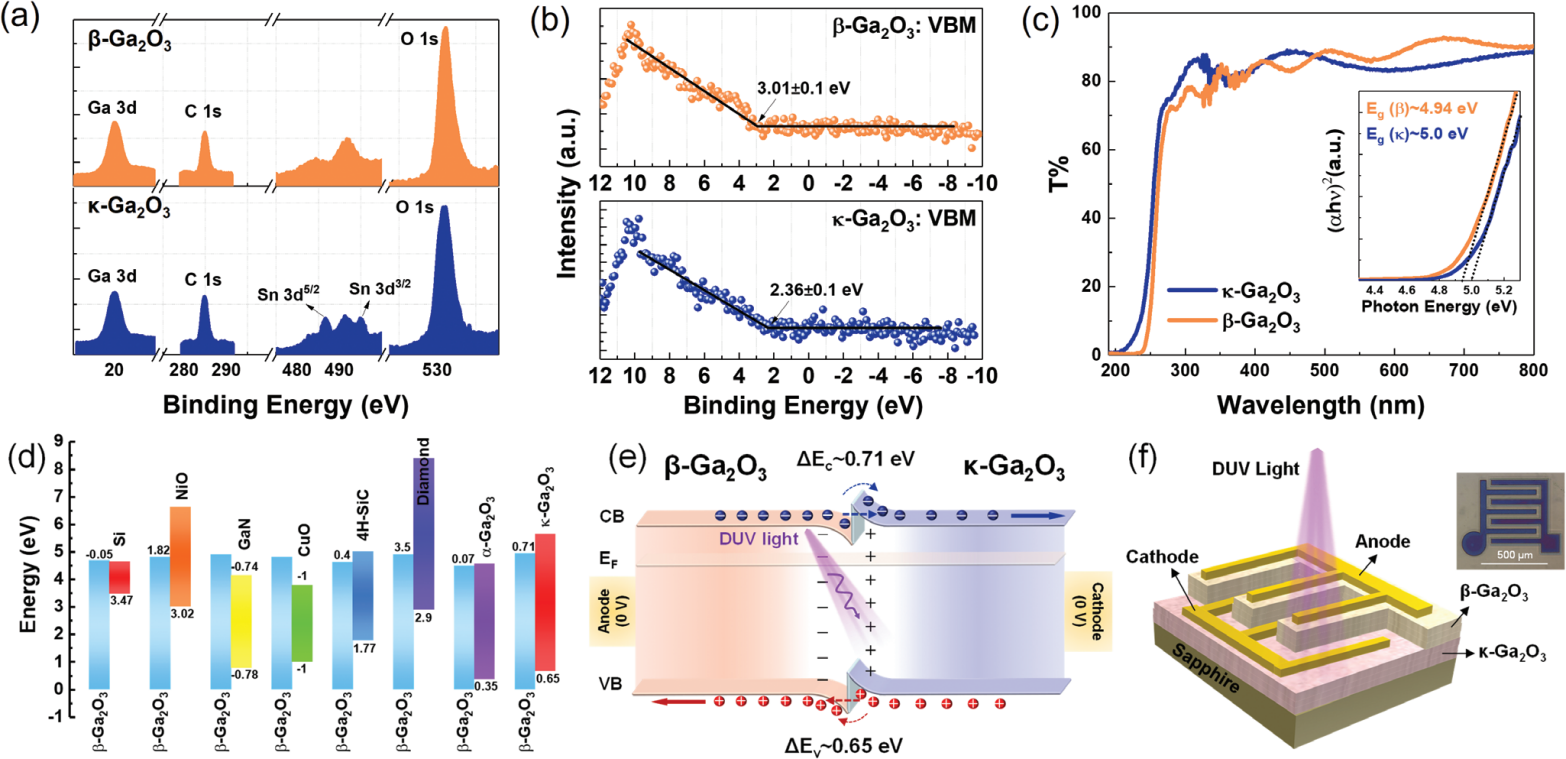
a) Core levels of Ga 3d, C 1s, Sn 3d, and O 1s of β‐Ga_2_O_3_ and κ‐Ga_2_O_3_. b) Valence band maximum (VBM) of β‐Ga_2_O_3_ and κ‐Ga_2_O_3_. c) Transmittance (*T*%) from UV–vis system. Inset is the Tauc plot figure of *T*%. d) Reported band alignment between p‐Si,^[^ ^28^ ^]^ p‐NiO,^[^ ^29^ ^]^ p‐GaN,^[^ ^30^ ^]^ p‐CuGaO_2_,^[^ ^31^ ^]^ p‐SiC,^[^ ^32^ ^]^ diamond,^[^ ^33^ ^]^ α‐Ga_2_O_3_,^[^ ^18b^ ^]^ and β‐Ga_2_O_3_. The last one is the alignment of β‐Ga_2_O_3_/κ‐Ga_2_O_3_ in this study. e) Schematic band diagram of β‐Ga_2_O_3_/κ‐Ga_2_O_3_ junction under zero bias. f) Schematic figure of fabricated β‐Ga_2_O_3_/κ‐Ga_2_O_3_ quasivertical PD. Inset is the top‐view microscope image of the real fabricated device.

In **Figure** **3**a, the binding energy of Ga 3d and O 1s core levels in β‐Ga_2_O_3_ is 20.01 and 531.11 eV, respectively, while in κ‐Ga_2_O_3_, Ga 3d and O 1s core levels are 20.07 and 531.07 eV, respectively. Notably, the core level positions of Ga 3d and O 1s in both β‐Ga_2_O_3_ and κ‐Ga_2_O_3_ remain consistent within an error margin of 0.06 eV. The VBM values in Figure 3b for β‐Ga_2_O_3_ and κ‐Ga_2_O_3_ are determined to be 3.01 ± 0.1 and 2.36 ± 0.1 eV, respectively. These VBM values were calculated by linearly extrapolating the leading edge of the respective valence band photoelectron spectrum to the baseline, following a previously described method.^[^ ^37^ ^]^ Using Equation (2), Δ*E*_v_ is calculated to be ≈0.65 eV. It is important to note that the core level and VBM positions have been aligned by referencing the C 1s peak at a binding energy of 284.8 eV.^[^ ^38^ ^]^

In Figure 3c, both the *T*% and its corresponding Tauc plot for β‐Ga_2_O_3_ and κ‐Ga_2_O_3_ are presented. The optical bandgap values for β‐Ga_2_O_3_ and κ‐Ga_2_O_3_ are determined to be 4.94 and 5.0 eV, respectively. These values are consistent with previously reported results.^[^ ^19^, ^20^ ^]^ Utilizing Equation (3), Δ*E*_c_ is calculated to be 0.71 eV. Thus, a type‐II band alignment for the β‐Ga_2_O_3_/κ‐Ga_2_O_3_ heterojunction with Δ*E*_c_ and Δ*E*_v_ of ≈0.71 and 0.65 eV, respectively, is experimentally shown. The experimentally derived band alignment is in agreement with previous findings in **Table** **1**, specifically a Δ*E*_c_ and Δ*E*_v_ of 0.71 and 0.63 eV, respectively, for the β‐Ga_2_O_3_/κ‐Ga_2_O_3_ junction according to the type‐II alignment of β‐Ga_2_O_3_/AlN and type‐I alignment of κ‐Ga_2_O_3_/AlN junctions.^[^ ^19^, ^20^ ^]^

**Table 1:** Conduction band offset (Δ*E*_c_) and valence band offset (ΔE_v_) values from literature reports and experiment. β‐Ga_2_O_3_/κ‐Ga_2_O_3_ (est) are estimated as Δ*E*_c_ and Δ*E*_v_ values based on the reported β‐Ga_2_O_3_/AlN^[^ ^19^ ^]^ and κ‐Ga_2_O_3_/AlN^[^ ^20^ ^]^ band alignment. β‐Ga_2_O_3_/κ‐Ga_2_O_3_ (exp) are measured Δ*E*_c_ and Δ*E*_v_ values in this study.

	*∆E*_c_ [eV]	*∆E*_v_ [eV]
β‐Ga_2_O_3_/AlN^[^ ^19^ ^]^	1.75	0.55
κ‐Ga_2_O_3_/AlN^[^ ^20^ ^]^	1.04	−0.08
β‐Ga_2_O_3_/κ‐Ga_2_O_3_ (est)	≈0.71	≈0.63
β‐Ga_2_O_3_/κ‐Ga_2_O_3_ (exp)	≈0.71	≈0.65

One promising application of this type‐II PHJ is the detection of solar‐blind DUV signals, particularly in the self‐powered mode. Self‐powered Ga_2_O_3_ PDs operate by relying on an electric field near the junction, for example, a pn junction, to separate photogenerated electrons and holes effectively and quickly without requiring an external bias. However, the lack of p‐type Ga_2_O_3_ necessitated the use of a different p‐type semiconductor. As shown in Figure 3d, various materials such as p‐Si,^[^ ^28^ ^]^ p‐NiO,^[^ ^22^, ^29^ ^]^ p‐GaN,^[^ ^30^ ^]^ p‐CuGaO_2_,^[^ ^31^ ^]^ p‐SiC,^[^ ^32^ ^]^ and diamond^[^ ^33^ ^]^ have been combined with n‐type Ga_2_O_3_ to form pn junctions, enabling a self‐powered photoresponse under DUV illumination without the need for an external bias. However, these materials possess different energy bandgaps, resulting in undesired absorption in the energy bands outside the solar‐blind region. Typically, when using a Ga_2_O_3_/GaN junction for light detection, a dual‐wavelength or broadband response is observed, covering the UVC to UVA regions.^[^ ^39^ ^]^ This may limit the Ga_2_O_3_ PD's capacity and their inherent sensitivity to solar‐blind DUV light. Therefore, the demonstrated type‐II single crystalline β‐Ga_2_O_3_/κ‐Ga_2_O_3_ PHJ which features an interfacial electrical field and similar absorption edges may present a favored performance for self‐powered DUV light detection.

As depicted in Figure 3e, in the β‐Ga_2_O_3_/κ‐Ga_2_O_3_ PHJ, β‐Ga_2_O_3_ and κ‐Ga_2_O_3_ are aligned to achieve a unified Fermi energy level in thermal equilibrium. This alignment results in downward and upward bending of energy levels near the surfaces of β‐Ga_2_O_3_ and κ‐Ga_2_O_3_, respectively. Simultaneously, a built‐in electric field is established from κ‐Ga_2_O_3_ to β‐Ga_2_O_3_ at the β‐Ga_2_O_3_/κ‐Ga_2_O_3_ interface. When exposed to DUV light, a significant number of electron–hole pairs are generated. Even in the absence of an external bias, these generated electrons and holes, which possess certain mobility, can be extracted to κ‐Ga_2_O_3_ and β‐Ga_2_O_3_, respectively, owing to the effect of the built‐in electric field. This leads to self‐powered performance of the device. To assess the photoresponse performance of the proposed β‐Ga_2_O_3_/κ‐Ga_2_O_3_ device, a quasivertical PD was fabricated, as shown in Figure 3f. Details regarding the fabrication process and parameters can be found in the Experimental Section. For comparing the self‐powered performance, additional bare β‐Ga_2_O_3_ and κ‐Ga_2_O_3_ samples were fabricated under the same conditions.

**Figure 4:**
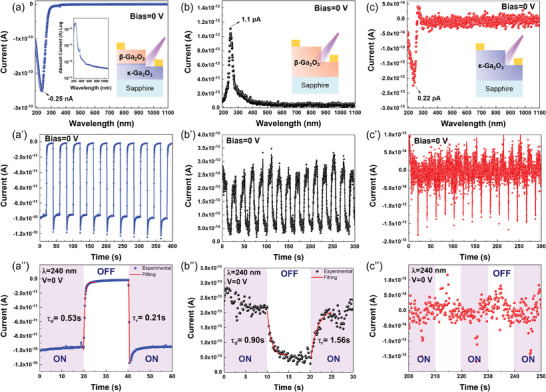
a–c) Photoresponse spectrum under zero bias, a′–c′) time‐dependent photocurrent under 240 nm illumination, aʺ–cʺ) fitted time‐dependent photocurrent under 240 nm illumination for β‐Ga_2_O_3_/κ‐Ga_2_O_3_ PHJ PD, β‐Ga_2_O_3_ PD, and κ‐Ga_2_O_3_ PD, respectively. Insets in (a), (b), and (c) contain the schematic figures of the fabricated β‐Ga_2_O_3_/κ‐Ga_2_O_3_ PHJ PD, β‐Ga_2_O_3_ PD, and κ‐Ga_2_O_3_ PD, respectively.

**Figure** **4** illustrates the photoresponse characteristics of the fabricated β‐Ga_2_O_3_/κ‐Ga_2_O_3_ PHJ PD, β‐Ga_2_O_3_ PD, and κ‐Ga_2_O_3_ PD. Under zero bias, samples (a), (b), and (c) exhibited photoresponses in the DUV region, as indicated by sharp peak currents. It is important to note that the anode was probed above the mesa for all samples during the measurements. For the β‐Ga_2_O_3_/κ‐Ga_2_O_3_ junction PD in (a), under zero bias, the peak current can reach −0.25 nA with DUV light illumination. This is attributed to the type‐II band alignment of the β‐Ga_2_O_3_/κ‐Ga_2_O_3_ junction, which features a built‐in electric field in the depletion region. This electric field automatically separates the electrons and holes generated under DUV illumination, leading to a photocurrent even in the absence of an external bias. Many studies have utilized Schottky‐type electrodes, such as Ni, Au, Pt, and graphene, on Ga_2_O_3_ to achieve self‐powered photodetection (Table S3, Supporting Information). However, in this work, the Ohmic contact characteristics of Ti/Au on both β‐Ga_2_O_3_ and κ‐Ga_2_O_3_ (Figure S6, Supporting Information) eliminate the possibility of a Schottky‐barrier‐induced electric field. Specifically, as shown in the band diagram in Figure 3e, the generated electrons flow to the κ‐Ga_2_O_3_ side, while the holes flow to the β‐Ga_2_O_3_ side under zero bias, driven by the built‐in electric field. This resulted in a negative photocurrent. The relationship between the photocurrent/responsivity and incident light intensity under zero bias is shown in Figure S7 (Supporting Information).

Repetitive measurements were performed at an on/off interval of 20 s to assess the response time and stability of the PD sample, as shown in Figure 4a′. When the shutter was alternated between the ON and OFF states, the photocurrent exhibited a distinct difference, even in the absence of a bias. This distinct photoswitching response curve indicated the reproducibility and stability of the PD. The response (rise) and recovery (decay) edges obtained within a single on–off cycle are shown in Figure 4aʺ. These edges can be fitted to an exponential relaxation equation, as shown in Equation (4)

(4) $$I=I_{0}+Ae^{-t/\tau }$$

where, *I* is the photocurrent, *I*_0_ is the steady‐state current, and *A* is the amplitude constant of the fitted model. Moreover, *t* is the time and *τ* is the fitted decay or rise component.^[^ ^40^ ^]^

Based on the fitting results, the response edge exhibited a rise time of 0.21 s, while the recovery edge demonstrated a decay time of 0.53 s, as calculated from Equation (4). The rise and decay times were influenced by two main factors. First, the on/off state of the interband optical transition plays a role in contributing to the carrier concentration. Second, the carrier trapping and releasing processes associated with the defects in the Ga_2_O_3_ film also affect these times. Some studies have demonstrated that the photoresponse time can be improved through postannealing,^[^ ^40^ ^]^ the use of a phototransistor,^[^ ^41^ ^]^ and Ar‐plasma treatment.^[^ ^42^ ^]^

In Figure 4aʹ, a notable observation is the significant current overshoot at the rising edges. This phenomenon has been previously documented in several studies.^[^ ^43^ ^]^ This occurs because under zero external bias, the built‐in potential is not sufficient to quickly drive the photogenerated carriers to the electrode. Consequently, the photocurrent decayed to a steady value when excess carriers recombined after a short period. It has been demonstrated that applying an external bias and increasing the temperature can eliminate this overshoot.^[^ ^44^ ^]^

Figure 4b,bʹ,bʺ,c,cʹ,cʺ depicts the PD performance for bare β‐Ga_2_O_3_ and κ‐Ga_2_O_3_ samples, both fabricated under the same conditions. Under zero bias, sharp photocurrent peaks in the DUV region were evident for both samples. However, this photocurrent is extremely low, ≈1.1 pA for β‐Ga_2_O_3_ (Figure 4b) and 0.22 pA for κ‐Ga_2_O_3_ (Figure 4c). Detecting such a low photocurrent poses a significant challenge because of the low signal‐to‐noise ratio. Ideally, the bulk Ga_2_O_3_ film under zero bias should exhibit a balanced photocurrent similar to the dark‐current level, because both electrodes have the same metal/semiconductor interface. Nevertheless, the presence of grain boundaries with potential differences and inhomogeneous defect distributions in the Ga_2_O_3_ epitaxial film may contribute to the separation of photogenerated electron–hole pairs, resulting in a self‐powered photocurrent under DUV illumination.^[^ ^45^ ^]^ In the case of β‐Ga_2_O_3_ in Figure 4bʹ,bʺ, a deformed on/off photocurrent curve can be observed during repetitive measurements, indicating a prolonged response time, with rise/decay times of 1.56 and 0.9 s, respectively. This suggests a limited capability for electron–hole separation, with the long response time potentially influenced by intrinsic defects and the presence of diffused Si impurities. Conversely, κ‐Ga_2_O_3_ in Figure 4cʹ,cʺ exhibits an ultralow photocurrent signal that significantly overlaps with the noise current, signifying negligible self‐powered photodetectivity. This can be attributed to the improved crystallinity of the κ‐Ga_2_O_3_ film compared to β‐Ga_2_O_3_, resulting in minor grain boundaries^[^ ^46^ ^]^ (i.e., larger grain size, as shown in Table S4 in the Supporting Information) and fewer intrinsic defects.^[^ ^47^ ^]^ The low dark current observed in the bare κ‐Ga_2_O_3_ PD also indicated a compensation effect from the amphoteric Si in the target (Figure S5, Supporting Information).

**Table** **2** provides a comparison of the PD performance of all three samples under zero bias. The β‐Ga_2_O_3_/κ‐Ga_2_O_3_ junction PD exhibits a photocurrent approximately three orders of magnitude higher than that of the κ‐Ga_2_O_3_ PD and β‐Ga_2_O_3_ PD, highlighting its superior self‐powered functionality. It also demonstrates a high on/off ratio of 580.8 when illuminated with DUV light (*λ* = 240 nm) or in the dark condition (*λ* = 1100 nm from the spectrum), resulting in a high responsivity of 17.8 mA W^−1^. In comparison to the bare β‐Ga_2_O_3_ and bare κ‐Ga_2_O_3_ PDs, the zero‐bias responsivity was ≈324 and 1369 times higher, respectively. The high on/off ratio and responsivity underscore the high sensitivity and capability of the proposed PD to detect ultraweak DUV signals. The responsivity (*R*) was calculated as follows

(5) $$R=\frac{I_{\mathrm{photo}}-I_{\mathrm{dark}}}{D\times S}$$

where *I*_photo_, *I*_dark_, *D*, and *S* are the photocurrent, dark current, illumination power density, and exposed area, respectively. Power density *D* is ≈11.3 µW cm^−2^ and exposure area *S* is ≈0.119 mm^2^.

**Table 2:** Comparison table of PD performance for β‐Ga_2_O_3_/κ‐Ga_2_O_3_ PHJ PD, β‐Ga_2_O_3_ PD, and κ‐Ga_2_O_3_ PD under zero bias.

	Bias [V]	|*I*_photo_| [A]	|*I*_photo_/*I*_dark_|	Responsivity [mA W^−1^]	D* [Jones]	EQE [%]	Rise time [s]	Decay time [s]
β‐Ga_2_O_3_/κ‐Ga_2_O_3_ PD	0	2.40 × 10^−10^	580.8	17.8	1.69 × 10^10^	9.20	0.21	0.53
β‐Ga_2_O_3_ PD	0	7.89 × 10^−13^	18.8	0.055	1.65 × 10^8^	0.0287	1.56	0.9
κ‐Ga_2_O_3_ PD	0	1.87 × 10^−13^	26.7	0.013	9.73 × 10^7^	0.0069	NA	NA

The detectivity (D*) of the PD, according to the signal‐to‐background noise ratio, is defined as follows^[^ ^48^ ^]^

(6) $$D^{*}=\sqrt{\frac{S}{2qI_{\mathrm{dark}}}}R$$

The external quantum efficiency (EQE) can be used to characterize the conversion efficiency of photogenerated electrons under photon illumination^[^ ^49^ ^]^

(7) $$\mathrm{EQE}=\frac{hc}{q\lambda }R\times 100\%$$

where *h* is the Planck constant, *c* is the speed of light, and *λ* is the wavelength of the incident light.

The comparisons of *R*, D*, EQE, and response time in Table 2 all demonstrate that the β‐Ga_2_O_3_/κ‐Ga_2_O_3_ junction exhibits superior capabilities in separating DUV‐generated electron–hole pairs. This superior performance can be attributed to the strong internal electric field at the interface originating from the Ga_2_O_3_/Ga_2_O_3_ PHJ.

**Figure 5:**
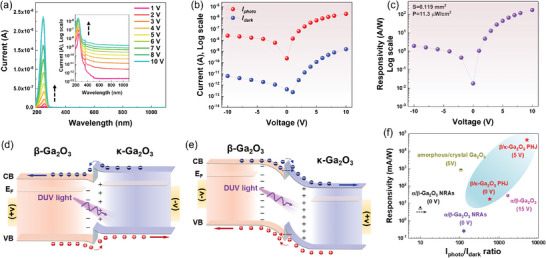
a) Photocurrent spectra of under different biases for β‐Ga_2_O_3_/κ‐Ga_2_O_3_ PHJ PD, inset is the log scale of the photocurrent spectra. b) Extracted photocurrent *I*_photo_ (*λ* = 240 nm) and dark current *I*_dark_ (*λ* = 1100 nm) under different biases. c) Responsivity of the β‐Ga_2_O_3_/κ‐Ga_2_O_3_ PHJ PD under different biases. Schematic diagram of the β‐Ga_2_O_3_/κ‐Ga_2_O_3_ PHJ under d) positive bias and e) negative bias. f) Self‐powered photoresponsivity and on/off ratio comparison of demonstrated β/κ‐Ga_2_O_3_ PHJ PD and reported Ga_2_O_3_/Ga_2_O_3_ mixed‐phase PD.^[^ ^18b,d,g,i^ ^]^

Furthermore, **Figure** **5**a shows the photocurrent spectra of the PD on linear and logarithmic scales with applied bias voltages ranging from 1 to 10 V and illumination wavelengths ranging from 190 to 1100 nm. The photocurrent increased as the applied bias voltage increased from 1 to 10 V, owing to improved carrier collection. The extended tail in the log scale of the spectrum (inset in Figure 5a) can be attributed to persistent photoconductivity (PPC), a phenomenon commonly observed in Ga_2_O_3_ PDs.^[^ ^50^ ^]^ PPC is associated with oxygen‐vacancy‐related traps, which can reduce the PD response speed and increase the PD recovery time, as photogenerated electrons are trapped and released in the trapping centers.^[^ ^51^ ^]^

The plot in Figure 5b shows the photocurrent (*I*_photo_) and dark current (*I*_dark_) under different bias conditions. For each bias, *I*_photo_ was over three orders of magnitude higher than *I*_dark_, indicating the high sensitivity of the PD in detecting DUV light. It can also be observed that under positive bias conditions, both *I*_photo_ and *I*_dark_ are higher than the values under negative bias conditions, indicating an apparent unilateral conductivity from −10 to 10 V. This phenomenon can be explained by the band diagrams shown in Figure 5d,e. Under a forward bias (Figure 5d), the external bias narrowed the internal depletion region and reduced the barrier height at the interface. Consequently, the electric field pushed the photogenerated carriers toward the electrode after crossing the barrier, resulting in a significantly enhanced current when the device was exposed to light. By contrast, under a reverse bias (Figure 5e), the strengthened internal electric field due to the applied bias severely bent down the energy band and accumulated photogenerated carriers at the interface. This leads to less efficient carrier transport and a lower photocurrent. However, some electrons and holes can still cross or tunnel through the barrier and be extracted by the electrode, forming a current, as shown in Figure 5e. This unilateral conductivity phenomenon from negative to positive bias also confirms the existence of interfacial electric field in this PHJ, and the rectification ratio is ≈87.8 for both photocurrent and responsivity at ±10 V. This rectification ratio is much higher than the deliberately fabricated n^+^/n^−^‐Ga_2_O_3_ Schottky junction PD (see Figure S8 in the Supporting Information for comparison), further confirming that the interfacial electric field existing at β/κ‐Ga_2_O_3_ junction is mainly induced by the type‐II band alignment.

Figure 5c presents the photoresponsivity of the proposed β‐Ga_2_O_3_/κ‐Ga_2_O_3_ junction PD under different biases. Because *I*_dark_ is three orders of magnitude smaller than *I*_photo_ and all configurations have the same effective *S*, the photoresponsivity is primarily determined by *I*_photo_. The photoresponsivity can reach ≈43.7 A W^−1^ at 5 V and 169 A W^−1^ at 10 V bias, indicating its significant potential for applications in solar‐blind detection. It is also noted that solar‐blind PDs based on mixed phase α/β‐Ga_2_O_3_ junctions have also been reported using the hydrothermal‐annealing method and sol–gel method.^[^ ^18b,d,g,i^ ^]^ However, the alleged α/β‐Ga_2_O_3_ junction is comprised of mixed phases with randomly distributed α/β/α/β interfaces and numerous grain boundaries, which is uncontrollable and may deteriorate the device performance. The benchmark results in Figure 5f show that, under zero bias, the demonstrated β‐Ga_2_O_3_/κ‐Ga_2_O_3_ PHJ PD exhibits significantly higher responsivity and on/off ratio than the previously reported α/β‐Ga_2_O_3_ nanorod array PDs, which contain multiple crystal orientations and unclear interfaces.^[^ ^18b,d^ ^]^ Under a 5 V bias, the β/κ‐Ga_2_O_3_ PHJ PD further excels, substantially outperforming both the amorphous/crystalline Ga_2_O_3_ PD and α/β‐Ga_2_O_3_ polycrystalline PD.^[^ ^18g,i^ ^]^ This enhanced benchmark highlights the effectiveness of the strong interfacial electric field in achieving efficient self‐powered solar‐blind detection. The detailed performance values are summarized in Table S5 (Supporting Information). Nevertheless, for the demonstrated β/κ‐Ga_2_O_3_ junction PD, several optimization strategies could be employed to further enhance performance, such as: i) reducing the thickness of the top β‐Ga_2_O_3_ layer to increase the absorption of the bottom κ‐Ga_2_O_3_ layer; ii) optimizing the interdigital pattern geometry;^[^ ^52^ ^]^ iii) improving the epitaxial quality of top β‐Ga_2_O_3_ layer.

## 3 Conclusion

In conclusion, this study experimentally demonstrates the β‐Ga_2_O_3_/κ‐Ga_2_O_3_ type‐II aligned “phase heterojunction” and shows its potential in applications where an interfacial depletion field is essential for efficient carrier separation, especially when operating in a self‐powered mode. HR‐TEM shows a distinct κ/β‐Ga_2_O_3_ phase heterojunction with a sharp, well‐defined interface and an ordered atomic arrangement. XPS measurement reveals a type‐II band alignment for the β‐Ga_2_O_3_/κ‐Ga_2_O_3_ heterojunction with Δ*E*_c_ and Δ*E*_v_ of 0.71 and 0.65 eV, respectively, implying a large interfacial electrical field. This interfacial electrical field allows for the separation of photogenerated electron–hole pairs without applying an external bias. Compared with the bare Ga_2_O_3_ PD, the PHJ PD exhibited approximately three orders of magnitude enhancement in photoresponsivity. The demonstrated PD has a responsivity, *I*_photo_/*I*_dark_ ratio, D*, EQE, and rise/decay time of 17.8 mA, 580.8, 1.69 × 10^10^ Jones, 9.2%, and 0.21/0.53 s, respectively, under zero bias, which are better than the reported Ga_2_O_3_/Ga_2_O_3_ mixed‐phase junction PD. Given that Ga_2_O_3_ exists in several polymorphs (α, β, γ, δ, ε, and κ phases), various PHJs involving different Ga_2_O_3_ phases could be further engineered to investigate the junction properties and advance electronic device applications. The κ/β phase heterojunction demonstrated in this work paves the way for future research into Ga_2_O_3_/Ga_2_O_3_ phase heterojunctions, offering new opportunities for exploration.

## 4 Experimental Section

### Material Growth and Characterization

As shown in Figure 1a, a κ‐Ga_2_O_3_ thin film was deposited onto a sapphire substrate using a PLD system, followed by the deposition of a β‐Ga_2_O_3_ thin film to create the β‐Ga_2_O_3_/κ‐Ga_2_O_3_ PHJ. The used targets had the following compositions: Ga_2_O_3_:SnO_2_:SiO_2_ (98.4:1.5:0.1, wt%) for κ‐Ga_2_O_3_ and unintentionally doped Ga_2_O_3_ for β‐Ga_2_O_3_, respectively. The deposition process involved ablation of the targets with a 300 mJ KrF excimer laser at a frequency of 5 Hz for 20 000 pulses for each target, resulting in a film thickness of ≈200 nm for each layer. The distance between the target and substrate was maintained at about 80 mm, while the substrate temperature was held at 680 °C. κ‐phase Ga_2_O_3_ could be stabilized at this temperature with good material quality. During the deposition of κ‐Ga_2_O_3_, the chamber was maintained under high vacuum conditions (<10^−6^ Torr). To attenuate the undesired etching of Ga_2_O_3_ caused by the formation of Ga_2_O in an oxygen‐less ambient, the deliberate incorporation of Sn could help to reoxidize Ga_2_O back into Ga_2_O_3_ and occupy octahedral lattice sites to induce the formation of κ‐phase Ga_2_O_3_. By contrast, the sequence deposition of β‐Ga_2_O_3_ took place in an O_2_ ambient with a pressure of 5 mTorr. The growth of β‐Ga_2_O_3_ under oxygen‐rich condition ensured a moderate growth rate and reduced the formation of oxygen vacancies. Additionally, two additional samples were prepared under the same growth conditions: bare β‐Ga_2_O_3_ and bare κ‐Ga_2_O_3_ on sapphire substrates, with each sample being subjected to 40 000 laser pulses.

The crystal structure properties of the deposited film were assessed using a Bruker D2 PHASER XRD system, employing a Cu tube (*λ* = 1.54184 Å) source at 30 kV. To estimate the optical bandgap of Ga_2_O_3_, transmittance measurements were conducted using a UV–vis spectrometer (Thermo Scientific Evolution 160). XPS analysis was carried out in a high vacuum environment using a Kratos Amicus XPS system, which featured a monochromatic Al Kα X‐ray source operating at 10 kV. This was performed to determine the band alignment at the junction between β‐Ga_2_O_3_ and κ‐Ga_2_O_3_. For the TEM analysis, a lamella was prepared using focused ion beam techniques within the FEI Helios G4 Scanning Electron Microscope (SEM) system. TEM imaging and FFT data were obtained using an FEI Titan Themis Z microscope system, with an acceleration voltage of 300 kV. Image processing was performed using Velox software. SIMS was employed for elemental depth profiling. This was accomplished by using a Dynamic SIMS system with an Ar acceleration energy of 4 kV.

### Device Fabrication and Characterization

All three aforementioned samples were fabricated using the same process flow used in this study. Following film deposition and material characterization, the samples underwent a cleaning procedure involving sequential rinsing with acetone, isopropanol, and deionized water, with each rinsing step lasting for more than 5 min. Subsequently, the samples were patterned using photolithography with positive photoresist AZ5214. Following patterning, all the samples were etched using an inductively coupled plasma‐reactive ion etching system under an Ar atmosphere. The etching process utilized a radio frequency (RF) power of 60 W and a BCl_3_ flow of 35 sccm for 6.5 min to create the mesa structure. The etching depth was ≈275 nm to ensure exposure of the underlying κ‐Ga_2_O_3_ layer. Subsequently, all samples were patterned once more for the deposition of Ti/Au (20/180 nm) electrodes using reactive sputtering. The resulting electrodes consisted of three pairs of interconnected parallel fingers, each with a width of 40 µm, a spacing gap of 40 µm, and a length of 450 µm. The effective *S* was 0.119 mm^2^. To establish an Ohmic contact between the electrode and Ga_2_O_3_, all samples underwent rapid thermal annealing at 600 °C for 1 min in an Ar ambient. Photoelectrical characteristics were measured using a Zolix DSR600‐X150‐200‐UV automated spectroradiometric measurement system. All measurements were conducted under ambient conditions within an optically sealed enclosure to prevent interference from surrounding light.

## Conflict of Interest

The authors declare no conflict of interest.

## Supporting information



Supporting Information

## Data Availability

The data that support the findings of this study are available from the corresponding author upon reasonable request.

## References

[1] a) X. Hou , X. Zhao , Y. Zhang , Z. Zhang , Y. Liu , Y. Qin , P. Tan , C. Chen , S. Yu , M. Ding , Adv. Mater. 2022, 34, 2106923; 10.1002/adma.202106923 34626038

[2] S. Pearton , J. Yang , P. H. Cary IV , F. Ren , J. Kim , M. J. Tadjer , M. A. Mastro , Appl. Phys. Rev. 2018, 5, 011301.

[3] M. J. Tadjer , Science 2022, 378, 724. 36395206 10.1126/science.add2713

[4] a) S. Stepanov , V. Nikolaev , V. Bougrov , A. Romanov , Rev. Adv. Mater. Sci. 2016, 44, 63;

[5] a) Q. Zhang , D. Dong , T. Zhang , T. Zhou , Y. Yang , Y. Tang , J. Shen , T. Wang , T. Bian , F. Zhang , ACS Nano 2023, 17, 24033; 38014834 10.1021/acsnano.3c08938

[6] a) R. Huang , H. Hayashi , F. Oba , I. Tanaka , J. Appl. Phys. 2007, 101, 063526;

[7] a) F. Mezzadri , G. Calestani , F. Boschi , D. Delmonte , M. Bosi , R. Fornari , Inorg. Chem. 2016, 55, 12079; 27934322 10.1021/acs.inorgchem.6b02244

[8] a) Y. Oshima , E. Ahmadi , Appl. Phys. Lett. 2022, 121, 260501;

[9] a) Y. Cai , K. Zhang , Q. Feng , Y. Zuo , Z. Hu , Z. Feng , H. Zhou , X. Lu , C. Zhang , W. Tang , Opt. Mater. Express 2018, 8, 3506;

[10] a) X. Lu , P. Yu , L. Zheng , S. Xu , M. Xie , S. Tong , Appl. Phys. Lett. 2003, 82, 1033;

[11] a) D. O. Scanlon , C. W. Dunnill , J. Buckeridge , S. A. Shevlin , A. J. Logsdail , S. M. Woodley , C. R. A. Catlow , M. J. Powell , R. G. Palgrave , I. P. Parkin , G. W. Watson , T. W. Keal , P. Sherwood , A. Walsh , A. A. Sokol , Nat. Mater. 2013, 12, 798; 23832124 10.1038/nmat3697

[12] a) J. Hou , C. Yang , Z. Wang , W. Zhou , S. Jiao , H. Zhu , Appl. Catal., B 2013, 142, 504;

[13] Y. H. Peng , Q. H. Liu , J. Q. Zhang , Y. Zhang , M. J. Geng , J. Q. Yu , J. Phys. Chem. C 2018, 122, 3738.

[14] Y. F. Zhang , S. Zhang , H. R. Sun , Z. Wang , F. X. Gao , J. W. Zhang , Z. Z. Liu , M. Fang , X. L. Tan , X. K. Wang , J. Environ. Chem. Eng. 2022, 10, 108781.

[15] Y. Katsumi , H. Gamo , J. Motohisa , K. Tomioka , ACS Appl. Mater. Interfaces 2024, 16, 30471. 38819142 10.1021/acsami.4c00147 PMC11182027

[16] a) R. Ji , Z. B. Zhang , Y. J. Hofstetter , R. Buschbeck , C. Hänisch , F. Paulus , Y. Vaynzof , Nat. Energy 2022, 7, 1170;

[17] a) T. Wang , W. Li , C. Ni , A. Janotti , Phys. Rev. Appl. 2018, 10, 011003;

[18] a) D. Guo , K. Chen , S. Wang , F. Wu , A. Liu , C. Li , P. Li , C. Tan , W. Tang , Phys. Rev. Appl. 2020, 13, 024051;

[19] H. Sun , C. Torres Castanedo , K. Liu , K.‐H. Li , W. Guo , R. Lin , X. Liu , J. Li , X. Li , Appl. Phys. Lett. 2017, 111, 162105.

[20] S. Krishna , Y. Lu , C.‐H. Liao , V. Khandelwal , X. Li , Appl. Surf. Sci. 2022, 599, 153901.

[21] a) Z. Zheng , X. Zu , Y. Zhang , W. Zhou , Mater. Today Phys. 2020, 15, 100262;

[22] a) X. Tang , Y. Lu , R. Lin , C.‐H. Liao , Y. Zhao , K.‐H. Li , N. Xiao , H. Cao , W. Babatain , X. Li , Appl. Phys. Lett. 2023, 122, 121101;

[23] a) S. Nakagomi , Y. Kokubun , J. Cryst. Growth 2012, 349, 12;

[24] M. Kracht , A. Karg , J. Schörmann , M. Weinhold , D. Zink , F. Michel , M. Rohnke , M. Schowalter , B. Gerken , A. Rosenauer , Phys. Rev. Appl. 2017, 8, 054002.

[25] a) M. Razeghi , J. Lee , L. Gautam , J.‐P. Leburton , F. H. Teherani , P. K. Amiri , V. P. Dravid , D. Pavlidis , Photonics 2021, 8, 578;

[26] β‐Ga2O3 (Ga2O3 ht) Crystal Structure: Datasheet from "PAULING FILE Multinaries Edition 2022" in SpringerMaterials, https://materials.springer.com/isp/crystallographic/docs/sd_0313635.

[27] κ‐Ga2O3 (Ga2O3 tf) Crystal Structure: Datasheet from "PAULING FILE Multinaries Edition 2022" in SpringerMaterials, https://materials.springer.com/isp/crystallographic/docs/sd_1638497.

[28] A. Atilgan , A. Yildiz , U. Harmanci , M. Gulluoglu , K. Salimi , Mater. Today Commun. 2020, 24, 101105.

[29] Y. Wang , C. Wu , D. Guo , P. Li , S. Wang , A. Liu , C. Li , F. Wu , W. Tang , ACS Appl. Electron. Mater. 2020, 2, 2032.

[30] P. Li , H. Shi , K. Chen , D. Guo , W. Cui , Y. Zhi , S. Wang , Z. Wu , Z. Chen , W. Tang , J. Mater. Chem. C 2017, 5, 10562.

[31] J. Shi , H. Liang , X. Xia , Q. Abbas , Appl. Surf. Sci. 2021, 569, 151010.

[32] J. Yu , L. Dong , B. Peng , L. Yuan , Y. Huang , L. Zhang , Y. Zhang , R. Jia , J. Alloys Compd. 2020, 821, 153532.

[33] Y.‐C. Chen , Y.‐J. Lu , C.‐N. Lin , Y.‐Z. Tian , C.‐J. Gao , L. Dong , C.‐X. Shan , J. Mater. Chem. C 2018, 6, 5727.

[34] E. Kraut , R. Grant , J. Waldrop , S. Kowalczyk , Phys. Rev. Lett. 1980, 44, 1620.

[35] a) F. Grunthaner , P. Grunthaner , Mater. Sci. Rep. 1986, 1, 65;

[36] a) J. Mickevičius , M. Andrulevicius , O. Ligor , A. Kadys , R. Tomašiūnas , G. Tamulaitis , E. Pavelescu , J. Phys. D: Appl. Phys. 2019, 52, 325105;

[37] S. A. Chambers , T. Droubay , T. C. Kaspar , M. Gutowski , J. Vac. Sci. Technol., B: Microelectron. Nanometer Struct. 2004, 22, 2205.

[38] P. Swift , Surf. Interface Anal. 1982, 4, 47.

[39] a) Y. Han , Y. Wang , S. Fu , J. Ma , H. Xu , B. Li , Y. Liu , Small 2023, 19, 2206664; 10.1002/smll.202206664 36683220

[40] D. Guo , Z. Wu , Y. An , X. Guo , X. Chu , C. Sun , L. Li , P. Li , W. Tang , Appl. Phys. Lett. 2014, 105, 023507.

[41] Z. Han , H. Liang , W. Huo , X. Zhu , X. Du , Z. Mei , Adv. Opt. Mater. 2020, 8, 1901833.

[42] L. Qian , H. Liu , H. Zhang , Z. Wu , W. Zhang , Appl. Phys. Lett. 2019, 114, 113506.

[43] a) X. Xu , J. Chen , S. Cai , Z. Long , Y. Zhang , L. Su , S. He , C. Tang , P. Liu , H. Peng , Adv. Mater. 2018, 30, 1803165; 10.1002/adma.201803165 30160338

[44] M. Zhang , Z. Liu , L. Yang , J. Yao , J. Chen , J. Zhang , W. Wei , Y. Guo , W. Tang , J. Phys. D: Appl. Phys. 2022, 55, 375106.

[45] a) D. Liu , F.‐J. Liu , J. Zhang , Z.‐X. Sa , M.‐X. Wang , S. P. Yip , J.‐C. Wan , P.‐S. Li , Z.‐X. Yang , J. Electron. Sci. Technol. 2023, 21, 100196;

[46] a) C.‐C. Yen , A. K. Singh , P.‐W. Wu , H.‐Y. Chou , D.‐S. Wuu , Mater. Today Adv. 2023, 17, 100348;

[47] a) T. K. Oanh Vu , D. U. Lee , E. K. Kim , J. Alloys Compd. 2019, 806, 874;

[48] X. Gong , M. Tong , Y. Xia , W. Cai , J. S. Moon , Y. Cao , G. Yu , C.‐L. Shieh , B. Nilsson , A. J. Heeger , Science 2009, 325, 1665. 19679770 10.1126/science.1176706

[49] D. Ma , J. Zhao , R. Wang , C. Xing , Z. Li , W. Huang , X. Jiang , Z. Guo , Z. Luo , Y. Li , ACS Appl. Mater. Interfaces 2019, 11, 4278. 30623664 10.1021/acsami.8b19836

[50] a) S. Hao , M. Hetzl , F. Schuster , K. Danielewicz , A. Bergmaier , G. Dollinger , Q. Sai , C. Xia , T. Hoffmann , M. Wiesinger , J. Appl. Phys. 2019, 125, 105701;

[51] a) H. Zhou , L. Cong , J. Ma , B. Li , M. Chen , H. Xu , Y. Liu , J. Mater. Chem. C 2019, 7, 13149;

[52] Z. Cai , X. He , K. Wang , X. Hou , Y. Mei , L. Ying , B. Zhang , H. Long , Small Methods 2024, 8, 2301148. 10.1002/smtd.202301148 38072623

