# Supplementary material for: κ/β‐Ga2O3 Type‐II Phase Heterojunction
Source: Adv Mater. 2025 Jan 13;37(8):2406902. doi: 10.1002/adma.202406902 (PMC11854863; doi:10.1002/adma.202406902)
Supplement: Supplementary file 1 — Supporting Information [file ADMA-37-2406902-s001.pdf]

ADVANCED MATERIALS

Supporting Information

for *Adv. Mater.*, DOI 10.1002/adma.202406902

# $\kappa/\beta$-$Ga_2O_3$ Type-II Phase Heterojunction

*Yi Lu, Patsy A. Miranda Cortez, Xiao Tang, Zhiyuan Liu, Vishal Khandelwal, Shibin Krishna and Xiaohang Li**

# Supporting Information

**κ/β-$Ga_2O_3$ Type-II Phase Heterojunction**

*Yi Lu, Patsy A Miranda Cortez, Xiao Tang, Zhiyuan Liu, Vishal Khandelwal, Shibin Krishna, Xiaohang Li* *

Advanced Semiconductor Laboratory
Electrical and Computer Engineering Program
Division of Computer, Electrical, and Mathematical Sciences and Engineering (CEMSE)
King Abdullah University of Science and Technology (KAUST)
Thuwal 23955-6900, Kingdom of Saudi Arabia
E-mail: xiaohang.li@kaust.edu.sa

Table S1: Summary of the crystal structures and parameters of $Ga_2O_3$ polymorphs.

| Phase | Crystal structure | Space group | Bandgap (eV) | Lattice constants (Å) | Specification | References |
|---|---|---|---|---|---|---|
| *α*-phase | Corundum | $R\bar{3}c$ | 5.2–5.3 | a = b = 4.98–5.04, c = 13.43–13.62 | Corundum structure analogous to *α*-$Al_2O_3$; largest bandgap | [1] |
| *β*-phase | Monoclinic | C2/m | 4.4–5.0 | a = 12.12–12.34, b = 3.03–3.04 c = 5.80–5.87 | Most thermodynamically stable; Native substrate available | [2] |
| *γ*-phase | Cubic defect spinel | $Fd\bar{3}m$ | 4.5–5.0 | a = 8.24–8.30 | Size-tunable emission in nanocrystal; Ferromagnetism property from Mn-doped γ-$Ga_2O_3$ | [1d, 3] |
| *δ*-phase | Bixbyite | $Ia\bar{3}$ | 4.8-5.0 | a = 9.4–10.0 | Synthesized using Mist-CVD on β-$Fe_2O_3$ buffer layer | [3a, 4] |
| *ε*-phase | Hexagonal | $P6_3mc$ | 4.5–5.0 | a = 5.12, b = 8.79, c = 9.41 | Metastable; Ferroelectric property | [1d, 3a, 5] |
| *κ*-phase | Orthorhombic | $Pna2_1$ | 4.6–5.0 | a = 5.05–5.06, b = 8.69–8.70 c = 9.28–9.31 | Metastable phase; Exhibit ferroelectric property | [5b, 5c, 6] |

Table S2 Summary of the reported "phase heterojunction" and their applications.

| Phase heterojunction | Synthesis method | Specification | Application | Year | References |
|---|---|---|---|---|---|
| Wurtzite GaN / Zincblende GaN | zincblende-GaN exist in wurzite-GaN as stacking faults | Type-II alignment with ~0.3 eV offset | Cubic GaN existing in hexagonal GaN grown on 6H-SiC from PL | 2003 | [7] |
| Wurtzite III-Nitrides / Zincblende III-Nitrides | Theoretical calculation | Different band offset between Wurtzite case and Zincblende case | DFT calculation result | 2020 | [8] |
| rutile $TiO_2$ / anatase $TiO_2$ | Theoretical calculation | Type-II alignment of 0.35/0.55 eV | Theorical calculation result | 2011 | [9] |
| rutile $TiO_2$ / anatase $TiO_2$ nanoparticles | Sol-gel and anneal to form rutile/anatase particles | Type-II alignment of ~0.4 eV | Enhanced electron-hole separation for photocatalysis | 2013 | [10] |
| rutile $TiO_2$ / anatase $TiO_2$ core-shell structure | ALD deposit $TiO_2$ on rutile $TiO_2$ NRs and anneal to rutile/anatase coreshell structure | Fast charge separation for electron/hole | PEC water splitting; $H_2$ evolution rates of 49.2 μmol $h^{-1}$ $cm^{-2}$ | 2019 | [11] |
| α-$Bi_2O_3$ / β-$Bi_2O_3$ | in situ hydrothermal process & post-heat treatment to form nanowire | Internal electrical field on the interface | Enhanced visible-light photocatalysis | 2013 | [12] |
| α-$Bi_2O_3$ / β-$Bi_2O_3$ | Hydrothermal process & heating to form nanoparticles | Generation and separation of charges on the interface | Enhanced and synergistic photocatalytic activity on degradation of toxic dye, Rhodamine-B under natural sunlight | 2018 | [13] |
| 0D $Bi_4MoO_9$ QDs / 2D $Bi_2MoO_6$ nanosheets | In situ hydrothermal process to form QDs/nanosheets | Efficient charge separation and transfer across the phase junction | Photocatalytic efficiency 13–23 times compared with pristine sample | 2018 | [14] |
| α-CdS / β-CdS | Heating precipitation-prepared CdS into hexagonal-cubic structure | Interfacial built-in electric field | Photocatalytic removal of U(VI) in water | 2022 | [15] |
| wurtzite InP / zincblende InP | Selective-area growth wurtzite InP nanowires on zinclende InP substrate using MOCVD | Type-II band discontinuity across the junction | GAA transistors; moderate current modulation with low SS. | 2024 | [16] |
| γ-$CsPbI_3$ / β-$CsPbI_3$ | solution deposition of β-$CsPbI_3$ and vapour deposition of the γ-$CsPbI_3$ | Increase built-in potential; enhance absorption | 20.1% solar cell efficiency | 2022 | [17] |
| γ-$CsPbI_3$ / β-$CsPbI_3$ | dynamic-hot-air method for depositing β-$CsPbI_3$ and then thermal evaporation of γ-$CsPbI_3$ | Type-II alignment for effective internal charge separation | 21.5% solar cell efficiency | 2023 | [18] |

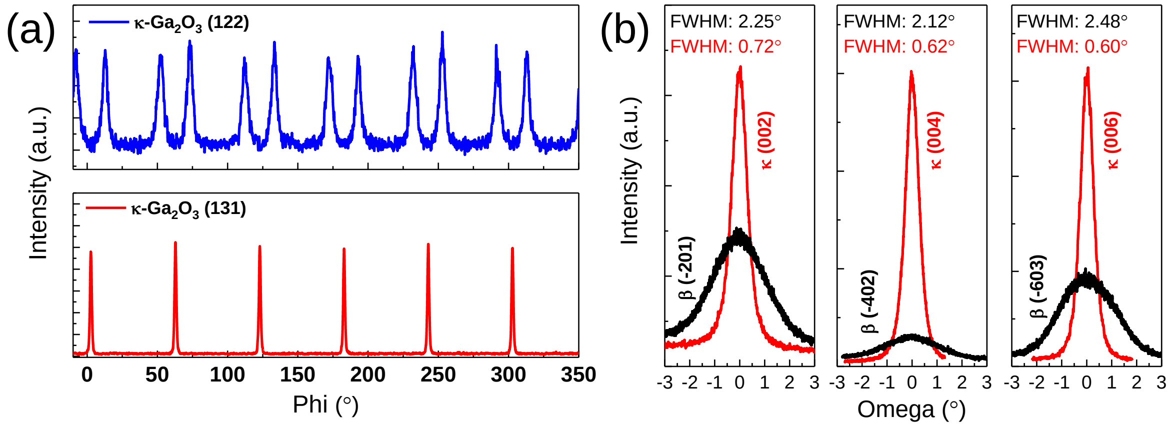


Figure S1. (a) XRD asymmetric ϕ scans for κ-$Ga_2O_3$ (122) and (131) planes. (b) XRD rocking curve and FWHM of β-$Ga_2O_3$ and κ-$Ga_2O_3$ on sapphire substrates.

The scan parameters for (122) plane were set as follows: ω = 16.66°, 2θ = 33.32°, and ψ = 54.63°, with a ϕ scan range from -10° to 350°. For the (131) plane, the XRD asymmetric ϕ scan was performed with ω, 2θ, and ψ positions of 18.5°, 37.00°, and 74.84°, respectively. These observations indicate the presence of an orthorhombic structure, confirming the formation of the orthorhombic κ-$Ga_2O_3$ phase rather than the hexagonal ε-phase. [6b, 19]

The lattice mismatch between the κ and β phases of $Ga_2O_3$ could be calculated below:

$$b_{\beta-Ga_2O_3} = 3.047\ \text{Å}$$

$$b_{\kappa-Ga_2O_3} = 8.702\ \text{Å}$$

$$\kappa\text{-}Ga_2O_3\langle 010\rangle // \beta\text{-}Ga_2O_3\langle 010\rangle = (1-\frac{8.702}{3\times 3.047})\times 100\% = 4.82\%$$

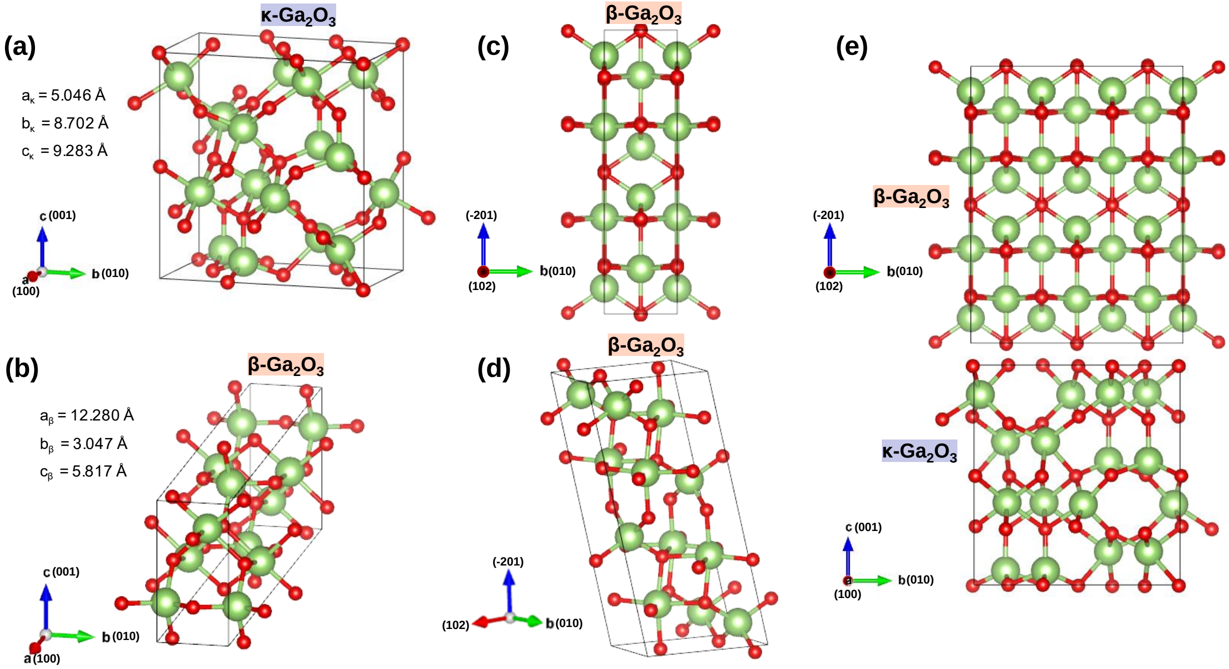


Figure S2. Unit cell for (a) κ-$Ga_2O_3$ and (b) β-$Ga_2O_3$. XRD and TEM data indicate that the κ-$Ga_2O_3$ thin film grew along the (001) direction, while β-$Ga_2O_3$ grew along the (-201) direction. In (d), β-$Ga_2O_3$ is oriented towards the (-201) direction, with orthogonal planes at (102) and (010). By rotating the unit cell into (c), the size of the lattice parameter b can be clearly identified. (e) illustrates the lattice mismatch calculation between three consecutive β-$Ga_2O_3$ unit cells along the (010) direction and one unit cell of κ-$Ga_2O_3$ along the same direction.

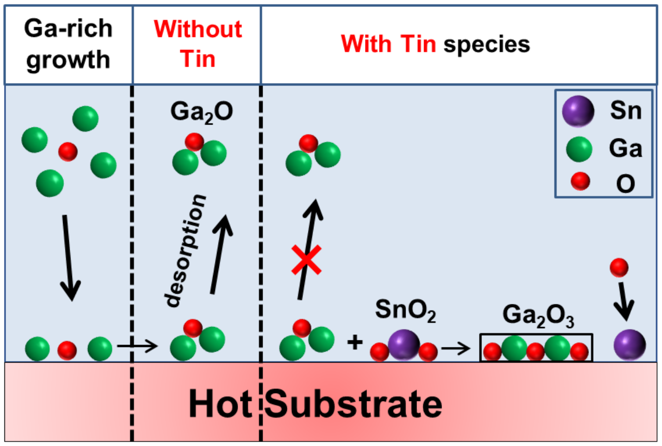


Figure S3. Growth mechanism of κ-$Ga_2O_3$ using Sn elements as a catalyst.

The Sn element as a catalyst contributes to two key aspects in forming κ-phase $Ga_2O_3$[20]: (i) Sn-assisted growth of $Ga_2O_3$ and (ii) the catalytic effect of Sn on phase formation.

$Ga_2O_3$ synthesis is typically conducted in an oxygen ambient to prevent undesirable reactions and etching of the film. In the absence of oxygen, the already-deposited $Ga_2O_3$ can react with excess Ga, forming $Ga_2O$ and leading to suboxide formation and film etching. The relevant reactions are as follows:

$$2\ Ga(g) + 3\ O(g) \rightarrow 1\ Ga_2O_3(s)$$

$$4\ Ga(g) + 1\ Ga_2O_3(s) \rightarrow 3\ Ga_2O\ (g)$$

However, in the presence of Sn on the surface, the etching process can be significantly attenuated. Sn adatoms react with $Ga_2O$, undergoing reduction to form SnO or metallic Sn, while the $Ga_2O$ is simultaneously oxidized to $Ga_2O_3$. This interaction between Sn and $Ga_2O$ helps protect the $Ga_2O_3$ film from excessive etching, thereby allowing the growth of $Ga_2O_3$ even under high vacuum conditions. This process can be described by the following reactions:

$$Ga_2O + 2SnO_2 \leftrightharpoons Ga_2O_3 + 2SnO \quad \Delta G^0 = -93.7kJ/mol$$
$$Ga_2O + 2SnO \leftrightharpoons Ga_2O_3 + 2Sn \quad \Delta G^0 = -117.7kJ/mol$$
$$Ga_2O + SnO_2 \leftrightharpoons Ga_2O_3 + Sn \quad \Delta G^0 = -105.7kJ/mol$$

The negative values of the standard Gibbs free energy change ($\Delta G^0$) for the reactions involving Sn adatoms indicate that these reactions are thermodynamically favorable and likely to occur. The presence of Sn species, such as SnO and $SnO_2$, can serve as oxygen sources that promote the oxidation of $Ga_2O$, leading to the formation of $Ga_2O_3$. When Sn adatoms are present, the reduced Sn species on the right side of the reaction can be reoxidized, regenerating $SnO_2$ and SnO.

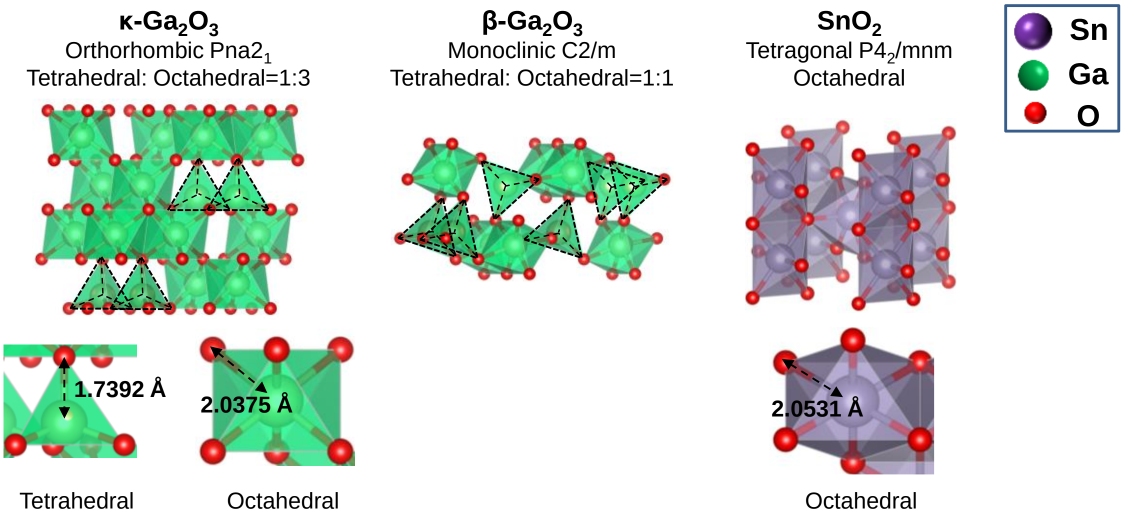


Figure S4. Unit cell coordination details and bond lengths of κ-$Ga_2O_3$, β-$Ga_2O_3$, and $SnO_2$.

Moreover, β-$Ga_2O_3$ has a tetrahedral/octahedral coordination ratio of 1:1, whereas the κ-$Ga_2O_3$ exhibits a 1:3 ratio between tetrahedral and octahedral coordinated metal atoms. Additionally, $SnO_2$ is characterized by octahedral coordination. In this context, Sn ions, which naturally prefer octahedral coordination with oxygen atoms, are more inclined to occupy the octahedral positions within the κ-$Ga_2O_3$ lattice. Besides, the bond length in $SnO_2$ is 2.0531 Å, while in $Ga_2O_3$, the bond lengths are approximately 1.7392 Å for tetrahedral positions and 2.0375 Å for octahedral positions. This disparity in bond lengths makes the octahedral lattice sites in $Ga_2O_3$ more favorable for Sn atoms to occupy.[21] Therefore, when Sn is present in the system, its preference for octahedral coordination and the longer bond lengths in $SnO_2$ facilitate the preferential κ-phase $Ga_2O_3$ formation.

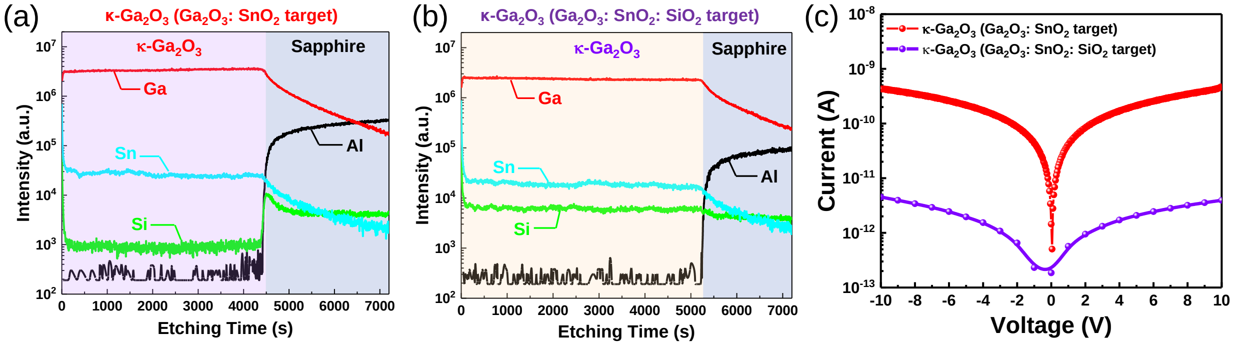


Figure S5. Elemental depth profiles obtained from dynamic SIMS for films grown using targets of (a) $Ga_2O_3$:$SnO_2$ (98.5%:1.5%, wt.) and (b) $Ga_2O_3$:$SnO_2$:$SiO_2$ (98.4%:1.5%:0.1%, wt.). The difference between these targets is the presence or absence of Si. Both samples were grown on c-plane sapphire substrates with same growth condition for consistent comparison. In (b), Si intensity is significantly higher in κ-$Ga_2O_3$, confirming its incorporation into the film. (c) IV characteristics of κ-$Ga_2O_3$ films grown from targets with different compositions.

Table S3. Reported $Ga_2O_3$-based self-powered solar-blind PDs with Schottky-type metal contacts.

| Type | Materials | Responsivity (mA/W) | Rejection ratio | Response speed | Ref |
|---|---|---|---|---|---|
| Schottky Junction | Au/$\beta$-$Ga_2O_3$ | 0.01 | $R_{258}/R_{280}$ ~ 11<br>$R_{258}/R_{400}$ ~ 38 | \ | [22] |
| | Au/$\beta$-$Ga_2O_3$ | \ | $R_{241}/R_{280}$ ~ 280<br>$R_{241}/R_{400}$ ~ 1080 | \ | [23] |
| | Pt/Ge:$\beta$-$Ga_2O_3$ | 0.09 | $R_{230}/R_{350}$ ~ $10^4$ | \ | [24] |
| | Ni/Au/$\beta$-$Ga_2O_3$ | 1.4 | \ | 1.1/0.3 s | [25] |
| | Au/$Ga_2O_3$/Si | \ | \ | 32.2/78 ms | [26] |
| | Ni/Au/$\beta$-$Ga_2O_3$ | $9.78 \times 10^3$ | $R_{212}/R_{350}$ ~ $10^4$ | 5.19 µs | [27] |
| | Graphene/Sn:$\beta$-$Ga_2O_3$ | \ | \ | 0.62/0.67 s | [28] |
| | Ni/Au/ $\beta$-$Ga_2O_3$ | 1.78 | $R_{260}/R_{500}$ ~ $7.1 \times 10^3$ | \ | [29] |

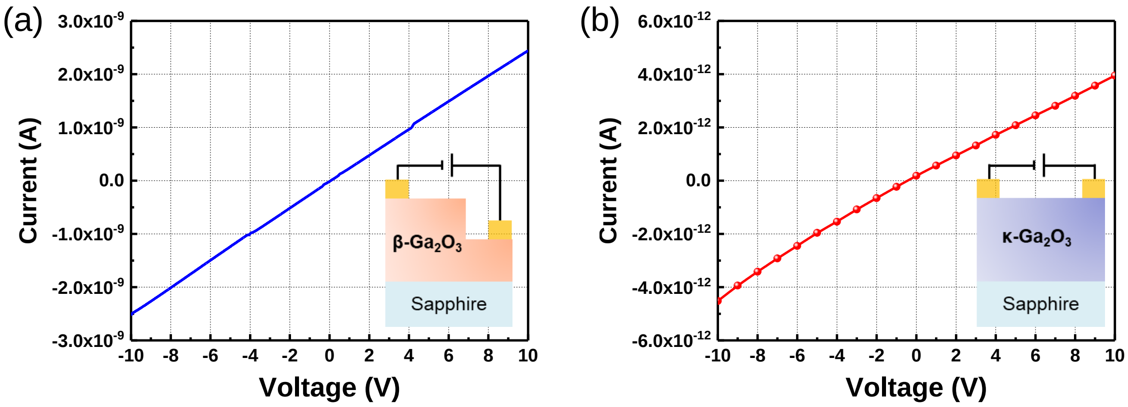


Figure S6. (a). IV curve measured from the β-$Ga_2O_3$ PD without illumination. (b) IV curve measured from the newly fabricated κ-$Ga_2O_3$ thin film without illumination.

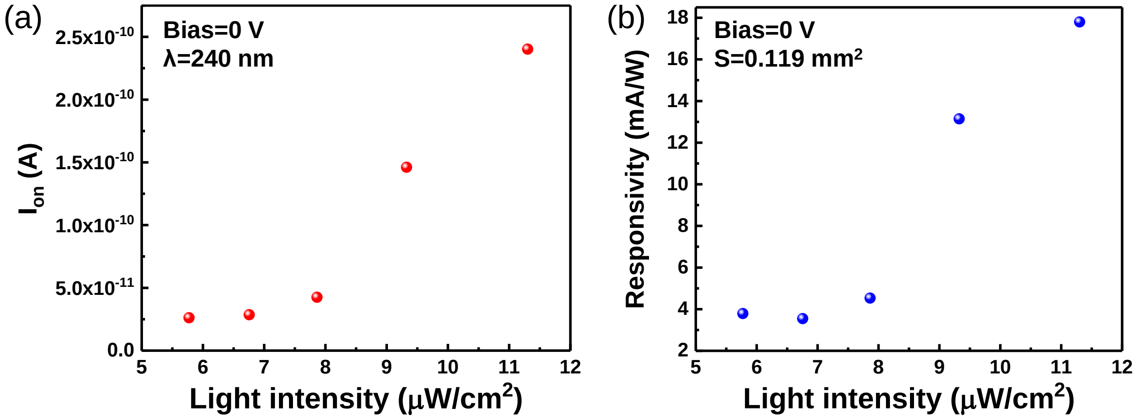


Figure S7. (a) Photocurrent ($I_{on}$) and (b) responsivity as a function of light power intensity under zero bias.

The photocurrent rises slowly at low light intensities and increases more rapidly at higher intensities. At low light intensities, the limited number of generated electron-hole pairs may become trapped at the interface or recombine with defect-related traps, resulting in relatively low photocurrent. At higher light intensities, the abundance of generated electron-hole pairs leads to higher photocurrent.

Table S4. Extracted thin film and device parameters from the three samples.

| Sample | 2θ (°)1st/2nd/3rd | FWHM (°)1st/2nd/3rd | Grain Size (nm) | $\lvert I_{photo}\rvert$ (A) @ 0 V | $\lvert I_{dark}\rvert$ (A) @ 0 V | Responsivity (mA/W) @ 0 V |
|---|---|---|---|---|---|---|
| β-$Ga_2O_3$ | 18.89°/38.28°/58.96° | 2.25/2.21/2.48 | ~3.54 | $7.89\times10^{-13}$ | $4.19\times10^{-14}$ | 0.055 |
| κ-$Ga_2O_3$ | 19.15°/38.80°/59.75° | 0.72/0.62/0.60 | ~11.14 | $1.87\times10^{-13}$ | $6.97\times10^{-15}$ | 0.013 |
| β-$Ga_2O_3$/κ-$Ga_2O_3$ | \ | \ | \ | $2.40\times10^{-10}$ | $4.13\times10^{-13}$ | 17.8 |

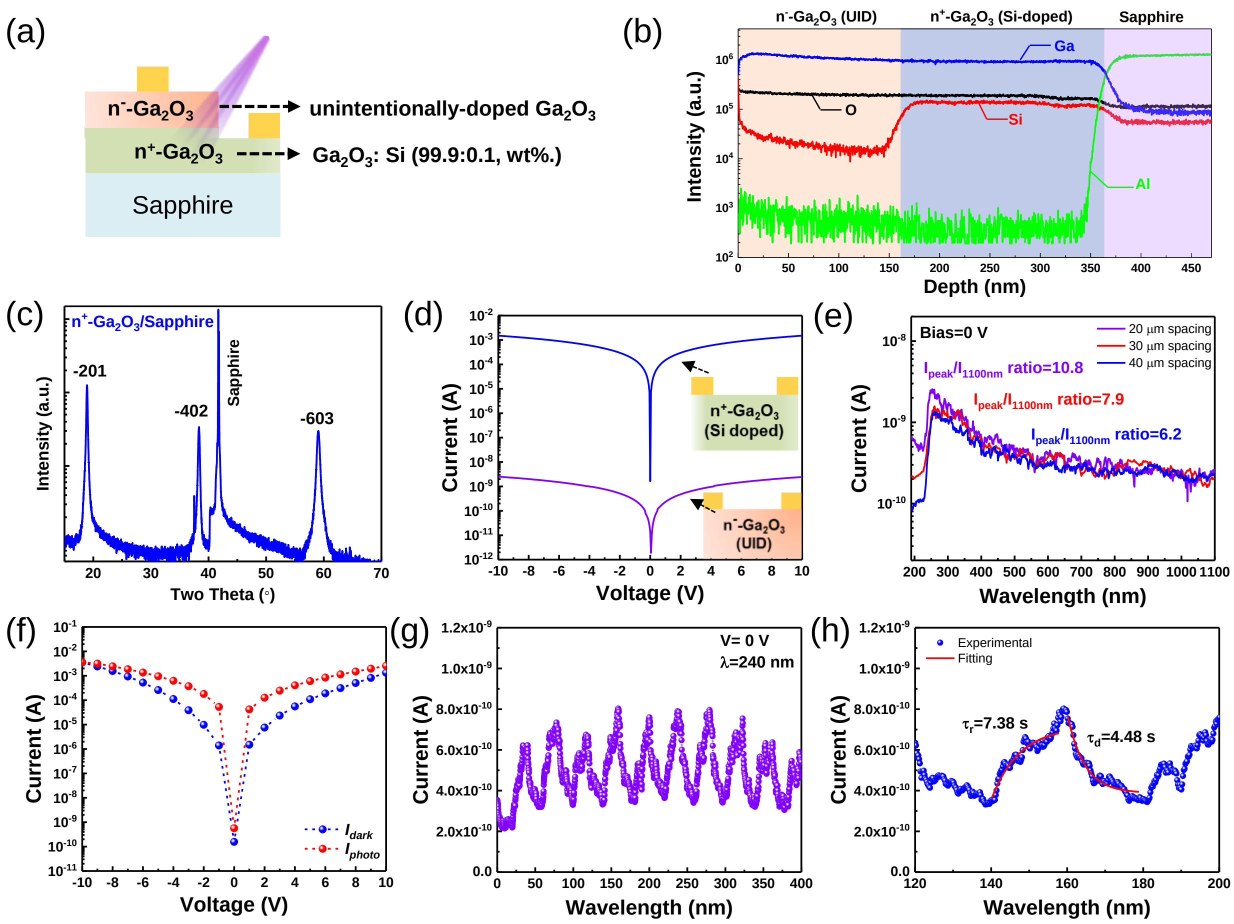


Figure S8. (a) Newly fabricated $n^+/n^-$-$Ga_2O_3$ Schottky junction PD; (b) SIMS elemental distribution for the $n^+/n^-$-$Ga_2O_3$ structure; (c) $n^+$-$Ga_2O_3$ XRD pattern; (d) IV characteristics of $n^+$ $Ga_2O_3$ and $n^-$-$Ga_2O_3$ films; (e) measured PD response spectra of $n^+/n^-$-$Ga_2O_3$ Schottky junction PD as a function of wavelength under zero bias; (f) photocurrent and dark current of $n^+/n^-$-$Ga_2O_3$ PD under applied bias. (g) time-dependent photocurrent and (h) fitted time-dependent photoresponse under 240 nm illumination and zero bias.

A $n^+/n^-$-$Ga_2O_3$ Schottky junction containing a Si-doped-$Ga_2O_3$/UID-$Ga_2O_3$ stack structure on sapphire was deliberately grew, shown in Figure S8 (a). The $n^+$-$Ga_2O_3$ layer was deposited using a $Ga_2O_3$: Si (99.9:0.1, wt%.) target, while the $n^-$-$Ga_2O_3$ layer was deposited using an unintentionally-doped (UID) $Ga_2O_3$ target. Figure S8(b) displays the distribution of Ga, O, Si, and Al in the $n^-$-$Ga_2O_3$/ $n^+$-$Ga_2O_3$/Sapphire stack structure, where enhanced Si concentration is evident in the $n^+$-$Ga_2O_3$ layer due to the intentional incorporation in the target. As seen in Figure S8 (c), the newly grown $n^+$-$Ga_2O_3$ film also exhibits a single β-phase orientation. The $n^+$-$Ga_2O_3$ layer exhibits a very high current under bias due to the ionized Si dopants, while the $n^-$-$Ga_2O_3$ shows six orders of magnitude lower current in Figure S8 (d). Both $n^+$ and $n^-$ layers show single crystal β-phase. Therefore, this stacked structure is comparable to an $n^+/n^-$-$Ga_2O_3$ Schottky junction.

Under zero bias, the $n^+/n^-$-$Ga_2O_3$ PD displays slight enhancement in the DUV region with an $I_{peak}/I_{1100nm}$ ratio between 6.2 and 10.8, which is significantly lower than the $I_{peak}/I_{1100nm}$ ratio of 580.8 observed in the β/κ-$Ga_2O_3$ phase junction PD. The rectification ratio of $n^+/n^-$-$Ga_2O_3$ junction at ±10 V is ~1.39, much lower than that of β/κ-$Ga_2O_3$ phase junction (~87.8), indicating a weak interfacial electrical field induced by this $n^+/n^-$-$Ga_2O_3$ Schottky junction. The $n^+/n^-$-$Ga_2O_3$ Schottky junction PD also exhibits severe persistent photoconductivity, with a rise and decay time of 7.38 and 4.48 s, respectively.

Table S5. Parameter list of reported $Ga_2O_3$/$Ga_2O_3$ junctions solar-blind PDs.

| Materials | Synthesis method | Voltage | $\lvert I_{photo}/I_{dark}\rvert$ | Responsivity (mA/W) | EQE (%) | Detectivity (Jones) | Response time | Ref |
|---|---|---|---|---|---|---|---|---|
| α-$Ga_2O_3$/β-$Ga_2O_3$ nanorod arrays | Hydrothermal & postannealing | 0 V | 127 | 0.26 | NA | $2.8\times10^{9}$ | 0.54/1.63 s | [30] |
| Amorphous $Ga_2O_3$/crystalline $Ga_2O_3$ film | Sputtering | 5 V | $10^{7}$ | 810 | 400 | $5.67\times10^{14}$ | 12 ns/19.6 μs | [31] |
| α-$Ga_2O_3$/β-$Ga_2O_3$ nanorod arrays | Hydrothermal & postannealing | 0 V | > 10 | 5.2 | NA | $1.45\times10^{14}$ | 0.12/0.29 s | [32] |
| α-$Ga_2O_3$/β-$Ga_2O_3$ polycrystalline film | Sol–gel | 15 V | 1664 | 28 | NA | $5.41\times10^{11}$ | 0.23/0.41 s | [33] |
| β-$Ga_2O_3$/κ-$Ga_2O_3$ film | Epitaxial growth | 0 V | 580.8 | 17.8 | 9.2 | $1.69\times10^{10}$ | 0.21/0.53 s | This work |
| β-$Ga_2O_3$/κ-$Ga_2O_3$ film | Epitaxial growth | 5 V | 5180 | 43686 | 22572 | $2.48\times10^{12}$ | 0.2/0.3 s | This work |

## References


[1] a)D. Tamba, O. Kubo, M. Oda, S. Osaka, K. Takahashi, H. Tabata, K. Kaneko, S. Fujita, M. Katayama, *Appl. Phys. Lett.* **2016**, 108, 251602; b)M. Oda, R. Tokuda, H. Kambara, T. Tanikawa, T. Sasaki, T. Hitora, *Appl. Phys. Express* **2016**, 9, 021101; c)H. Y. He, R. Orlando, M. A. Blanco, R. Pandey, E. Amzallag, I. Baraille, M. Rérat, *Phys. Rev. B* **2006**, 74, 195123; d)S. Yoshioka, H. Hayashi, A. Kuwabara, F. Oba, K. Matsunaga, I. Tanaka, *J. Phys.: Condens. Matter* **2007**, 19, 346211; e)M. Marezio, J. Remeika, *J. Chem. Phys.* **1967**, 46, 1862.

[2] a)J. A. Kohn, G. Katz, J. D. Broder, *Am. Mineral.* **1957**, 42, 398; b)J. Ahman, G. Svensson, J. Albertsson, *Acta Crystallogr., Sect. C: Cryst. Struct. Commun.* **1996**, 52, 1336; c)H. Y. He, R. Orlando, M. A. Blanco, R. Pandey, E. Amzallag, I. Baraille, M. Rérat, *Phys. Rev. B* **2006**, 74; d)X. L. Zhao, M. F. Ding, H. D. Sun, S. B. Long, *Semiconductors and Semimetals* **2021**, 107, 101.

[3] a)S. I. Stepanov, V. I. Nikolaev, V. E. Bougrov, A. E. Romanov, *Rev. Adv. Mater. Sci.* **2016**, 44, 63; b)R. Huang, H. Hayashi, F. Oba, I. Tanaka, *J. Appl. Phys.* **2007**, 101, 063526; c)Y. Teng, A. Ponchel, Z. K. Yang, J. Xia, *Adv. Mater.* **2014**, 26, 6238; d)D. Kisailus, J. H. Choi, J. C. Weaver, W. J. Yang, D. E. Morse, *Adv. Mater.* **2005**, 17, 314; e)C.-C. Huang, C.-S. Yeh, *New J. Chem.* **2010**, 34, 103.

[4] a)S. K. Ghosh, *ACS Omega* **2020**, 5, 25493; b)M. R. Karim, Z. Feng, J. M. Johnson, M. Zhu, J. Hwang, H. Zhao, *Cryst. Growth Des.* **2019**, 19, 1965; c)T. Kato, H. Nishinaka, K. Shimazoe, K. Kanegae, M. Yoshimoto, *ACS Appl. Electron. Mater.* **2023**, 5, 1715.

[5] a)P. Kroll, R. Dronskowski, M. Martin, *J. Mater. Chem.* **2005**, 15, 3296; b)Y. Arata, H. Nishinaka, D. Tahara, M. Yoshimoto, *Jpn. J. Appl. Phys.* **2020**, 59, 025503; c)F. Mezzadri, G. Calestani, F. Boschi, D. Delmonte, M. Bosi, R. Fornari, *Inorg. Chem.* **2016**, 55, 12079.

[6] a)I. Cora, F. Mezzadri, F. Boschi, M. Bosi, M. Čaplovičová, G. Calestani, I. Dódony, B. Pécz, R. Fornari, *CrystEngComm* **2017**, 19, 1509; b)M. Kneiß, A. Hassa, D. Splith, C. Sturm, H. Von Wenckstern, T. Schultz, N. Koch, M. Lorenz, M. Grundmann, *APL Mater.* **2019**, 7, 022516; c)J. Kim, D. Tahara, Y. Miura, B. G. Kim, *Appl. Phys. Express* **2018**, 11, 061101; d)K. Shimazoe, H. Nishinaka, Y. Arata, D. Tahara, M. Yoshimoto, *AIP Adv.* **2020**, 10, 055310.

[7] X. Lu, P. Yu, L. Zheng, S. Xu, M. Xie, S. Tong, *Appl. Phys. Lett.* **2003**, 82, 1033.

[8] Y. C. Tsai, C. Bayram, *ACS Omega* **2020**, 5, 3917.

[9] P. Deák, B. Aradi, T. Frauenheim, *J. Phys. Chem. C* **2011**, 115, 3443.

[10] a)D. O. Scanlon, C. W. Dunnill, J. Buckeridge, S. A. Shevlin, A. J. Logsdail, S. M. Woodley, C. R. A. Catlow, M. J. Powell, R. G. Palgrave, I. P. Parkin, G. W. Watson, T. W. Keal, P. Sherwood, A. Walsh, A. A. Sokol, *Nat. Mater* **2013**, 12, 798; b)R. Su, R. Bechstein, L. Sø, R. T. Vang, M. Sillassen, B. Esbjörnsson, A. Palmqvist, F. Besenbacher, *J. Phys. Chem. C* **2011**, 115, 24287.

[11] N. Wei, Y. Liu, M. Feng, Z. Li, S. Chen, Y. Zheng, D. Wang, *Appl. Catal. B* **2019**, 244, 519.

[12] J. Hou, C. Yang, Z. Wang, W. Zhou, S. Jiao, H. Zhu, *Appl. Catal. B* **2013**, 142-143, 504.

[13] K. K. Bera, R. Majumdar, M. Chakraborty, S. K. Bhattacharya, *J. Hazard. Mater.* **2018**, 352, 182.

[14] Y. H. Peng, Q. H. Liu, J. Q. Zhang, Y. Zhang, M. J. Geng, J. Q. Yu, *J. Phys. Chem. C* **2018**, 122, 3738.

[15] Y. F. Zhang, S. Zhang, H. R. Sun, Z. Wang, F. X. Gao, J. W. Zhang, Z. Z. Liu, M. Fang, X. L. Tan, X. K. Wang, *J. Environ. Chem. Eng.* **2022**, 10, 108781.
[16] Y. Katsumi, H. Gamo, J. Motohisa, K. Tomioka, *ACS Appl. Mater. Interfaces* **2024**, 16, 30471.
[17] R. Ji, Z. B. Zhang, Y. J. Hofstetter, R. Buschbeck, C. Hänisch, F. Paulus, Y. Vaynzof, *Nat. Energy.* **2022**, 7, 1170.
[18] S. S. Mali, J. V. Patil, J.-Y. Shao, Y.-W. Zhong, S. R. Rondiya, N. Y. Dzade, C. K. Hong, *Nat. Energy.* **2023**, 8, 989.
[19] D. Tahara, H. Nishinaka, S. Morimoto, M. Yoshimoto, *Appl. Phys. Lett.* **2018**, 112, 152102.
[20] a)Y. Cai, K. Zhang, Q. Feng, Y. Zuo, Z. Hu, Z. Feng, H. Zhou, X. Lu, C. Zhang, W. Tang, *Opt. Mater. Express* **2018**, 8, 3506; b)M. Kracht, A. Karg, J. Schörmann, M. Weinhold, D. Zink, F. Michel, M. Rohnke, M. Schowalter, B. Gerken, A. Rosenauer, *Phys. Rev. Appl.* **2017**, 8, 054002.
[21] J. B. Varley, J. R. Weber, A. Janotti, C. G. Van de Walle, *Appl. Phys. Lett.* **2010**, 97, 142106.
[22] X. Chen, K. Liu, Z. Zhang, C. Wang, B. Li, H. Zhao, D. Zhao, D. Shen, *ACS Appl Mater Interfaces* **2016**, 8, 4185.
[23] C. Yang, H. Liang, Z. Zhang, X. Xia, P. Tao, Y. Chen, H. Zhang, R. Shen, Y. Luo, G. Du, *RSC Adv.* **2018**, 8, 6341.
[24] F. Alema, B. Hertog, A. Osinsky, P. Mukhopadhyay, M. Toporkov, W. V. Schoenfeld, E. Ahmadi, J. Speck, *Oxide-Based Materials and Devices Viii* **2017**, 10105, 101051M.
[25] A. S. Pratiyush, S. Krishnamoorthy, S. Kumar, Z. Xia, R. Muralidharan, S. Rajan, D. N. Nath, *Jpn. J. Appl. Phys.* **2018**, 57, 060313.
[26] K. Arora, N. Goel, M. Kumar, M. Kumar, *ACS Photonics* **2018**, 5, 2391.
[27] Y. Xu, X. Chen, Y. Zhang, F. Ren, S. Gu, J. Ye, *IEEE Electron Device Lett.* **2020**, 41, 1.
[28] G. Kalita, R. D. Mahyavanshi, P. Desai, A. K. Ranade, M. Kondo, T. Dewa, M. Tanemura, *Phys. Status Solidi - Rapid Res. Lett.* **2018**, 12, 1800198.
[29] H. Sheoran, S. Fang, F. Z. Liang, Z. Huang, S. Kaushik, N. Manikanthababu, X. L. Zhao, H. D. Sun, R. Singh, S. B. Long, *ACS Appl. Mater. Interfaces* **2022**, 14, 52096.
[30] C. Wu, C. He, D. Guo, F. Zhang, P. Li, S. Wang, A. Liu, F. Wu, W. Tang, *Mater. Today Phys.* **2020**, 12, 100193.
[31] Y. Wang, W. Cui, J. Yu, Y. Zhi, H. Li, Z.-Y. Hu, X. Sang, E.-j. Guo, W. Tang, Z. Wu, *ACS Appl. Mater. Interfaces* **2019**, 11, 45922.
[32] H. Hu, L. Deng, Y. Zhu, C. Wu, D. Guo, S. Wang, *J. Alloys Compd.* **2023**, 945, 169307.
[33] M. Yu, C. Lv, J. Yu, Y. Shen, L. Yuan, J. Hu, S. Zhang, H. Cheng, Y. Zhang, R. Jia, *Mater. Today Commun.* **2020**, 25, 101532.
